# Supplementary material for: Inter-Synaptic Lateral Diffusion of GABAA Receptors Shapes Inhibitory Synaptic Currents
Source: Neuron. 2017 Jul 5;95(1):63–69.e5. doi: 10.1016/j.neuron.2017.06.022 (PMC5500312; doi:10.1016/j.neuron.2017.06.022)
Supplement: Document S1. Figures S1–S4 [file mmc1.pdf]

Neuron, Volume *95*

Supplemental Information

Inter-Synaptic Lateral Diffusion

of GABAA Receptors Shapes

Inhibitory Synaptic Currents

Emanuela de Luca, Tiziana Ravasenga, Enrica Maria Petrini, Alice Polenghi, Thierry Nieus, Stefania Guazzi, and Andrea Barberis

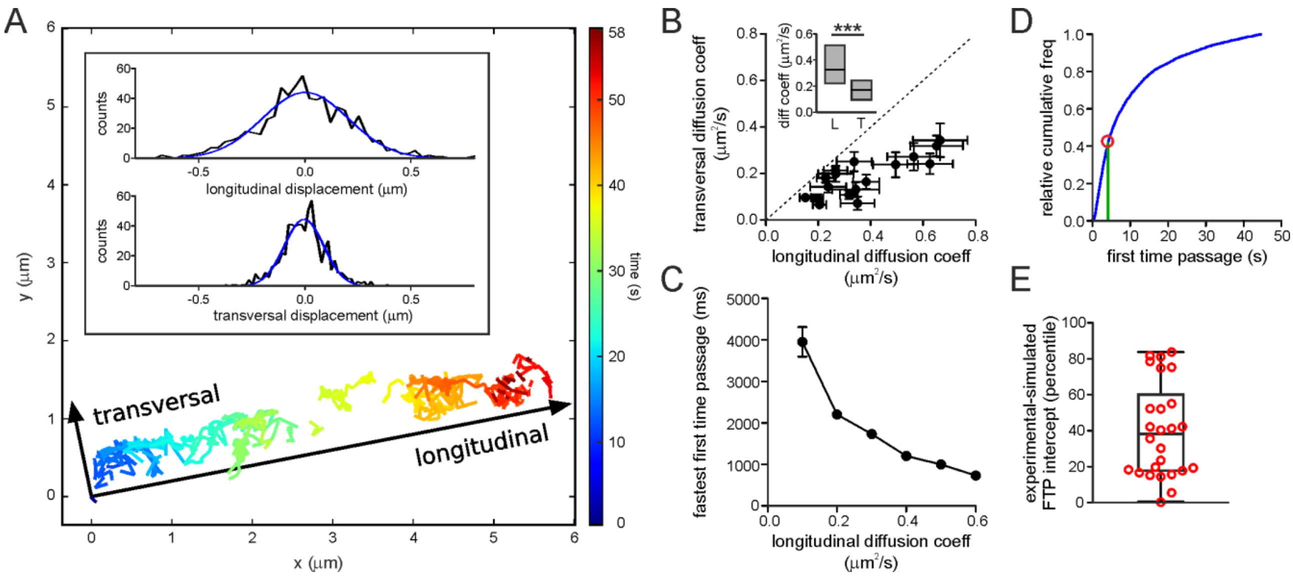

Figure S1. (Related to Figure 1) Analysis and model simulations of GABAA receptor diffusion in bounded conditions

(A) Representative trajectory of a GABAA receptor diffusing along a linear dendrite (time is represented in pseudocolors). The experimentally-recorded motion is decomposed along the longitudinal and transversal directions. Please note that blinkings are not represented, resulting in interrupted trajectory. (inset) The longitudinal and transversal displacements (black) are well fitted by a Gaussian curve (blue). Please note the much shorter displacement along the transversal direction.

(B) Longitudinal and transversal diffusion coefficients of the GABAA receptor trajectories (n=18 displacements along “mainly linear” portions of dendrites out of 25). Error bars correspond to confidence intervals. The dashed line shows identical diffusion coefficients in both the longitudinal and transversal components. Inset: Median diffusion coefficient of longitudinal (“L”) and transversal (“T”) displacements. “L”: median = 0.33 $\mu m^2 s^{-1}$, IQR=0.22÷0.67 $\mu m^2 s^{-1}$, $n_{simulated\ trajectories}$=18. “T”: median = 0.17 $\mu m^2 s^{-1}$, IQR=0.07÷0.24 $\mu m^2 s^{-1}$, $n_{simulated\ trajectories}$=18, p<0.001, Wilcoxon matched paired test U-test.

(C) Relationship between the fastest first time passage (f-FTP) and the longitudinal diffusion coefficient. The f-FTP values were obtained from the first percentile of each cumulative distribution (parameters dSYN=2 µm, TMAX=60 s). Data are presented as mean ± SEM.

(D) Representative example of FTP cumulative distribution of simulated inter-synaptic events identified in 5000 repetitions run to reproduce a single experiment (blue). In order to mimic experimental conditions, the longitudinal diffusion coefficient, the chance time and the inter-synaptic distance imposed in the simulations (see Methods) were those identified experimentally. The green line shows the inter-synaptic time quantified experimentally. The red circle highlights the intercept of the two lines, and represents the value of the percentile corresponding to the experimentally calculated inter-synaptic time. Please note that this plot refers to a single experiment.

(E) Raw data and superimposed boxplot (median and IQR range) of the “intercept values” between the inter-synaptic time and the FTP curves as described in D for all the experiments analysed (n=26). Please note that the values are nearly distributed around the 50th percentile.

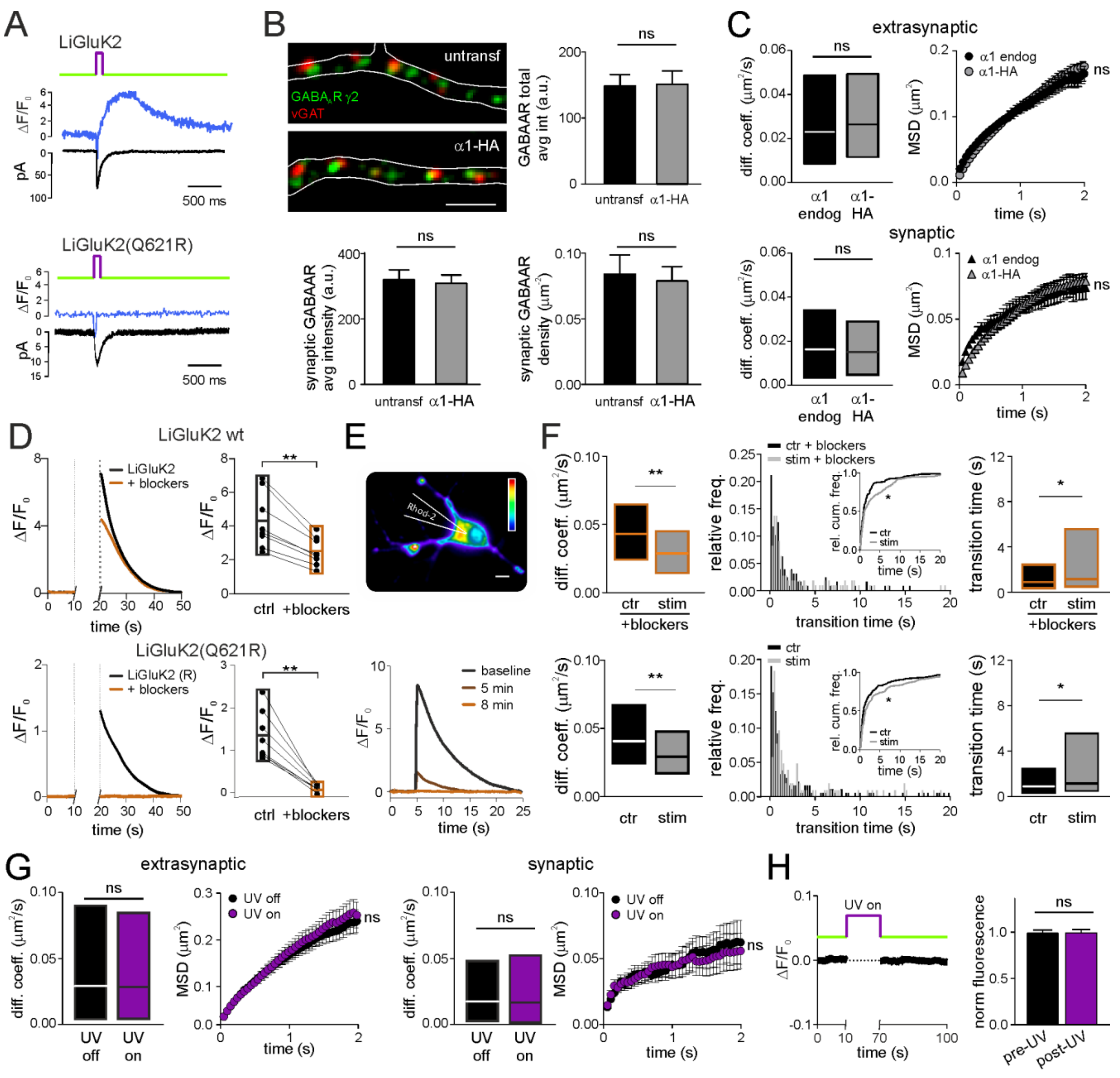

Figure S2. (Related to Figure 2) Regulation of intracellular calcium by light-gated glutamate receptor (LiGluK2) and VGCCs

(A) Representative relative changes ($\Delta F/F_0$) of Rhod-2 fluorescence (blue traces) and light-evoked currents (black traces) elicited by LiGluK2 (top) and LiGluK2(Q621R) (bottom) openings upon illumination with 380nm light. The above lines show the illumination protocol: green = 556nm and violet = 380nm. Please note that the illumination with 380 nm light (violet bars) causes an increase in intracellular calcium and consequently increases the Rhod-2 fluorescence intensity (blue traces) in LiGluK2- and not in LiGluK2(Q621R)-expressing neurons. Currents were obtained from the average of at least 10 traces.

(B) GABAAR incorporating the HA-α1 subunit show properties comparable to endogenous receptors. (top, left) Representative fluorescence images of GABAAR γ2 subunit immunoreactivity (green) along with the presynaptic marker vGAT (red) in untransfected and HA-α1–expressing neuron as indicated. Scalebar 5 μm.

(top right and bottom) Quantification of GABAARs total surface immunoreactivity and GABAARs synaptic cluster intensity and density in untransfected and HA-α1–expressing neurons (n=15 and 14, respectively, from 2 independent cultures, p>0.05, unpaired t-test).

(C) Comparable lateral mobility of endogenous and HA-tagged α1 subunits probed by SPT. (top, left) GABAAR diffusion coefficient at extrasynaptic compartments ($n_{trajectories}$: endogenous=320 in 32 neurons from 7 independent cultures; HA-α1= 609, in 54 neurons from 8 independent cultures; p>0.05, Mann–Whitney U-test). (top, right) Mean Square Displacement (MSD) vs time plots of endogenous ($n_{trajectories}$=325, in 32 neurons from 7 independent cultures) and HA-α1 tagged GABAARs ($n_{trajectories}$=609, in 54 neurons from 8 independent cultures, p>0.05, Student's t-test) at extrasynaptic compartments. (bottom, left) Diffusion coefficient of synaptic GABAARs ($n_{trajectories}$: endogenous=130, in 32 neurons from 7 independent cultures; HA-α1= 192, in 54 neurons from 8 independent cultures, p>0.05, Mann–Whitney U-test). (bottom, right) MSD vs time plots of endogenous ($n_{trajectories}$=158, in 32 neurons from 7 independent cultures) and HA-α1 GABAARs at synapses ($n_{trajectories}$=189, in 54 neurons from 8 independent cultures, p>0.05, Student's t-test).

(D) Contribution of VGCCs in the modulation of inter-synaptic displacements by LiGluK2 activation. Top: P/Q-, N- and L-type VGCCs account for ~ 40% of the calcium increase induced by LiGluK2 activation. (left) Representative examples of calcium transients following the opening of the LiGluK2 wt in control conditions (black) and after the application of P/Q-, N- and L-type VGCC blockers (ω-conotoxin MVIIC, 2μM and nifedipine, 10μM, respectively, hereafter indicated as "VGCCs blockers") (orange). (right) Matched peak calcium transients induced by LiGluK2 wt openings before (black) and after (orange) the application of VGCC blockers (n=8 neurons, p<0.01, paired Wilcoxon test). Bottom: Upon the activation of LiGluK2(Q621R), the calcium increase is ~ 70% less than that mediated by LiGluK2 wt, and is mainly accounted for by P/Q-, N- and L-type voltage-gated calcium channels. (left) Representative traces of calcium transients following the opening of the LiGluK2(Q621R) in control conditions (black) and after the application of the VGCC blockers (orange). (right) Matched peak calcium transients induced by LiGluK2(Q621R) opening before (black) and after (orange) the VGCCs block (n=8 neurons, P<0.01, paired Wilcoxon test).

(E) Pharmacological block of VGCCs. (top) Representative cultured hippocampal neuron expressing LiGluK2 loaded with Rhod-2 (shown in pseudocolors). Scale bar 5 μm. (bottom) Representative calcium transients evoked by cell depolarization (300ms, 0mV) in control condition (black), 5 minutes (brown) and 8 minutes (orange) after the application VGCC blockers. Note that at 5 minutes the calcium increase is only partially blocked, whereas it is completely abolished after 8 minutes.

(F) During LiGluK2 activation, VGCCs do not play a major role in the modulation of GABAA receptor inter-synaptic mobility. Top: Modulation of HA-GABAAR inter-synaptic transitions by LiGluK2 activation in the presence of VGCC blockers. (left) Inter-synaptic HA-GABAAR diffusion coefficient in the presence of VGCC blockers before (ctr, $n_{trajectories}$=72) and upon LiGluK2 activation ($n_{trajectories}$=63; in 17 neurons from 5 independent cultures, p<0.01, Mann–Whitney U-test). (middle) Histogram and cumulative distribution (inset) of inter-synaptic displacement time in the control (black) and during LiGluK2 activation (gray) upon VGCCs blockade. (n=129 and 110, respectively, in 17 neurons from 5 independent cultures, p<0.05, Kolmogorov Smirnov test). (right) HA-GABAAR median and IQR of inter-synaptic transition time in control (n=129) and upon stimulation with LiGluK2 in the presence of VGCC blockers (n= 110; p<0.05, Mann–Whitney U-test). Bottom: Same as above, without VGCC blockers. (left) Diffusion coefficient of inter-synaptic HA-GABAARs before (ctr, $n_{trajectories}$=97) and during LiGluK2 activation (stim, $n_{trajectories}$=66; p<0.01, Mann–Whitney U-test; 18

neurons from 5 independent cultures). (middle) Histogram and cumulative distribution (inset) of inter-synaptic displacement time in the control (black) and during LiGluK2 activation (gray). (n=185 and 104, respectively, in 18 neurons from 5 independent cultures, $p<0.05$, Kolmogorov Smirnov test). (right) HA-GABAAR median and IQR of inter-synaptic transition time in control (n=185) and upon stimulation with LiGluK2 (n= 104; $p<0.05$, Mann–Whitney U-test; 18 neurons from 5 independent cultures).

(G) UV illumination does not alter GABAAR lateral mobility. Diffusion coefficient (left) and MSD vs time plots (right) of HA-GABAAR illuminated with 490nm (UV off, black) or with 380nm (UV on, violet) light at extrasynaptic and synaptic compartments as indicated. Extrasynaptic diffusion coefficient, $n_{trajectories}$: UV off=263; UV on= 232, in 12 neurons from 2 independent cultures; $p>0.05$, Mann–Whitney U-test. MSD of extrasynaptic HA-GABAAR, $n_{trajectories}$: UV off=282, UV on=258, in 12 neurons from 2 independent cultures; $p>0.05$, Student's t-test. Diffusion coefficient of synaptic HA-GABAAR, $n_{trajectories}$: UV off=61; UV on = 61, in 12 neurons from 2 independent cultures, $p>0.05$, Mann–Whitney U-test. MSD of synaptic HA-GABAARs, $n_{trajectories}$: UV off=66; UV on=66, in 12 neurons from 2 independent cultures, $p>0.05$, Student's t-test.

(H) UV illumination does not alter intracellular calcium. (left) Representative relative change in Rhod-2 fluorescence ($\Delta F/F_0$) elicited by 60 s UV illumination of an untransfected neuron. The green and violet lines above show the illumination pattern as in panel A. (right) Normalized Rhod-2 fluorescence quantified before and after 60 s UV illumination (n=12, $p>0.05$, paired t test).

Unless otherwise stated, data are presented as mean values ± SEM. The boxplots indicate the median and interquartile range (IQR). Relative changes in Rhod-2 fluorescence are expressed as percentage units. ns, non significant; *, $p<0.05$; **, $p<0.01$.

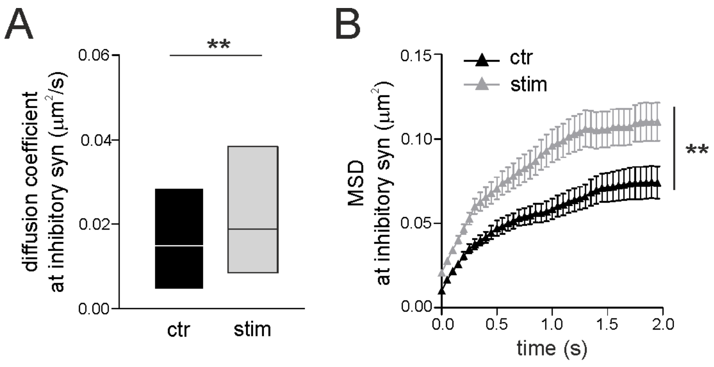

Figure S3. (Related to Figure 3) LiGluK2 activation modulates GABAAR diffusion at inhibitory synapses

(A) Diffusion coefficient of synaptic GABAAR for control (ctr) and 1 min LiGluK2 activation (stim) (median=0.014 $\mu m^2 s^{-1}$, IQR=0.006-0.033 $\mu m^2 s^{-1}$, $n_{trajectories}$=107; and median=0.021 $\mu m^2 s^{-1}$ IQR=0.010-0.046 $\mu m^2 s^{-1}$, $n_{trajectories}$=128, respectively, p<0.01; Mann–Whitney U-test; 28 neurons from 7 independent cultures).

(B) MSD versus time plot of GABAARs diffusing at inhibitory synapses (steady state: ctrl=0.075±0.009 μm^2, $n_{trajectories}$ 107 and stim=0.110±0.011 μm^2, $n_{trajectories}$ 128; 28 neurons from 7 independent hippocampal cultures, p<0.01, Student's t-test at steady state).

Data are presented as mean values ± SEM. The boxplots indicate the median and IQRs. **, p<0.01.

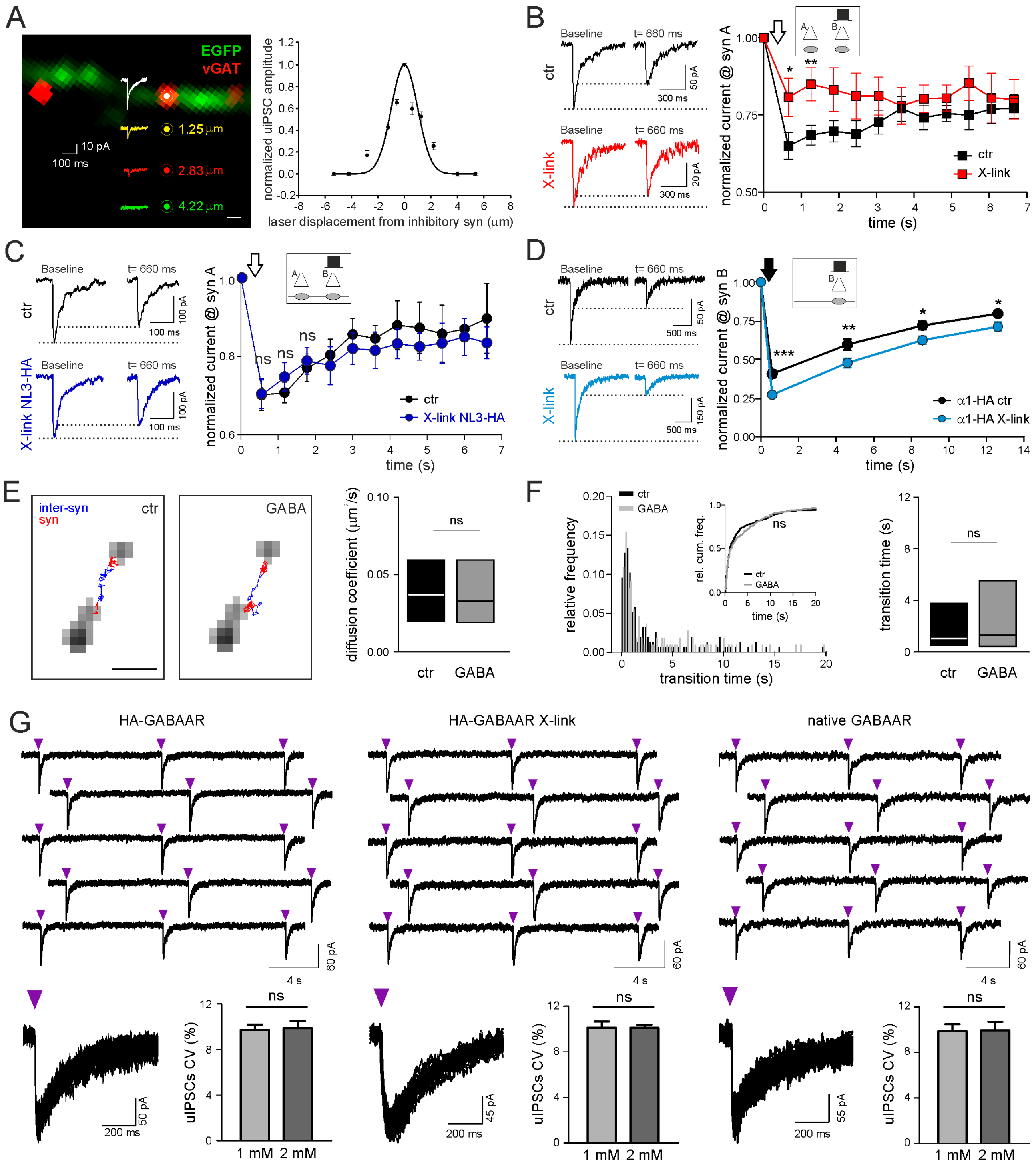

Figure S4. (Related to Figure 4) GABAA receptor desensitization by GABA uncaging and functional implications

(A) (left) Representative image of a EGFP-transfected neuron (green) with GABAergic synapses identified by live labelling with anti-vGAT-Oyster550 antibody (red). Scale bar: 500 nm. uIPSCs were elicited by laser pulses delivered in the synapse center (white) and progressively displaced away from the synapse (yellow: 1.25 μm; red: 2.83 μm; green: 4.22 μm). (right) Summary of the relationship between uIPSCs amplitude and the laser spot position with respect to the synapse center. The measured full width at half maximum (FWHM) was 2.34±0.24 μm (n=2).

(B) The inter-synaptic diffusion of endogenous GABAA receptors modulates inhibitory synaptic responses. (left) Representative traces of uIPSCs mediated by endogenous GABAA receptors in control (ctr, black) and after the X-link protocol (X-link, red), before and 660 ms after the delivery of a UV laser train pulse at an adjacent inhibitory synapse, according to the protocol shown in Figure 4A and schematized in the inset on the right. Dashed lines show the reduction in uIPSCs current amplitude before and after the train (i.e., desensitization). (right). Normalized recovery over time of uIPSCs recorded at synapse "A" after delivery of the UV laser train pulse at synapse "B" (arrow) in control (ctr, black, n= 18, from 2 independent neuronal cultures) and after the X-link protocol in untransfected neurons (X-link, red, n= 16, from 2 independent neuronal cultures). At 660 ms: ctr=0.65±0.04, n=18; X-link=0.81±0.06, n=16; $p<0.05$. unpaired t test.
(C) The X-link protocol does not affect the recovery of uncaging currents. (Left) Representative traces of uIPSCs recorded in control (ctr) and in Neuroligin3 X-link protocol (NL3) before (baseline) and 660 ms after the UV laser train of stimuli at an adjacent synapse. Dashed lines show the reduction in uIPSCs current amplitude before and after the train (i.e., desensitization). (right) Normalized uncaging current amplitude recovery of synapse B (see protocol schematization in the inset) over time after the application of the UV laser train at synapse A (arrow) in control (ctr-black, n=12) and upon Neuroligin3 X-link (NL3 blue n=14). Please note that the X-link protocol directed to the synaptic protein NL3 does not affect the time course of the uncaging current amplitude recovery (time point 660 ms: ctr=0.71±0.03, n_{values}=12 from 11 neurons and 5 independent hippocampal cultures; NL3=0.75±0.04, n_{values}=14 from 7 neurons and 3 independent hippocampal cultures; $p>0.05$, Mann–Whitney U-test).
(D) Repetitive GABA uncaging at inhibitory synapses renders GABAAR into the desensitized state. (left) Representative uncaging currents elicited before (left) and 660 ms after (right) the delivery of the UV laser train pulse in control conditions (ctr, black) and upon X-link (light blue). Please note that in these experiments the train is delivered at the same synapse tested (see protocol schematization in the inset). Dashed lines show the reduction in uIPSCs current amplitude before and after the train (i.e., desensitization). (right) Normalized uncaging current amplitude over time after delivery of the UV laser train pulse (plain arrow) in control (ctr, black, n= 8 from 3 independent neuronal cultures) and upon X-link (X-link, light blue, n= 14 from 4 independent neuronal cultures). At 660 ms: ctr=0.41±0.03, n=8; X-link=0.27±0.02, n=14; $p< 0.001$, Mann–Whitney U-test.
(E) Inter-synaptic displacements of desensitized GABAAR. (left) Reconstructed synaptic (red) and inter-synaptic (blue) trajectories of the same HA-GABAA receptor in control conditions and upon replacement of the recording solution with GABA 100 μM, a concentration that, being more than 5 times the GABA EC50, is expected to promote massive GABAA receptor desensitization. Live-immunolabelling of vGAT at inhibitory synapses is shown in gray. Scalebar 1 μm. (right) Diffusion coefficient of inter-synaptic HA-GABAAR in control (median=0.037 $\mu m^2 s^{-1}$, IQR=0.020-0.059, $n_{trajectories}$=95; GABA 100 μM (GABA), median=0.033 $\mu m^2 s^{-1}$, IQR=0.019-0.060, $n_{trajectories}$=65; 19 neurons from 5 independent cultures; $p>0.05$, Mann–Whitney U-test).
(F) (left) Histogram and cumulative distribution (inset) of HA-GABAAR inter-synaptic displacement time in the control (black, n=153) and upon solution exchange with GABA 100 μM (gray, n=109), $p>0.05$, Kolmogorov Smirnov test. (right) Inter-synaptic transition time of desensitized HA-GABAAR. Control: Median=1.05 s; IQR=0.50-3.78 s, n=153; GABA: Median=1.30 s; IQR=0.45-5.52 s, n=109; 19 neurons from 5 independent cultures; $p>0.05$, Mann–Whitney U-test. (G) Saturation of inhibitory synapses by GABA uncaging pulses. Top. Representative uncaging experiments aimed at probing synaptic saturation by quantification of synaptic

uIPSCs variability in neurons expressing HA-GABAAR (left), HA-GABAAR upon X-link (middle) and native GABAA receptors (right). Laser pulses were delivered every 10 s to uncage GABA 1 mM (violet arrowheads) at inhibitory synapses. (Bottom) Superimposed uIPSCs recorded in the experiment shown above and mean coefficient of variation (CV) of uIPSCs elicited by the laser photolysis of caged GABA 1 mM (9.73±0.47 %, n= 5) and 2 mM (9.88±0.62 %, n= 4, p>0.05, unpaired t test) in the indicated conditions, i.e. HA-GABAAR (left), HA-GABAAR upon X-link (middle) and native GABAA receptors (right). The unaffected CV values with a double concentration of caged GABA (2 mM) indicate the saturation of inhibitory synapses challenged with the uncaging pulses. In neuron expressing HA-GABAAR: $CV_{GABA\ 1\ mM}$ = 9.73±0.47 % (n= 5); $CV_{GABA\ 2\ mM}$ = 9.88±0.62 % (n= 4), p>0.05, unpaired t test. In neurons expressing HA-GABAAR upon receptor X-link: $CV_{GABA\ 1mM}$ = 10.13±0.52 (n=5); $CV_{GABA\ 2mM}$ = 10.11±0.27 (n=3), p>0.05, unpaired t test. In untransfected neurons expressing only native GABAARs: $CV_{GABA\ 1mM}$ = 9.87±0.62 (n=5); $CV_{GABA\ 2mM}$ = 9.95±0.75 (n=5), p>0.05, unpaired t test.

Unless otherwise stated, data are presented as mean values ± SEM. The boxplots indicate the median and IQRs. ns, non significant; *, p<0.05; **, p<0.01. ***, p<0.001.
