# Supplementary material for: Inter-Synaptic Lateral Diffusion of GABAA Receptors Shapes Inhibitory Synaptic Currents
Source: Neuron. 2017 Jul 5;95(1):63–69.e5. doi: 10.1016/j.neuron.2017.06.022 (PMC5500312; doi:10.1016/j.neuron.2017.06.022)
Supplement: Document S2. Article plus Supplemental Information [file mmc2.pdf]

Neuron

Report

Inter-Synaptic Lateral Diffusion of GABAA Receptors Shapes Inhibitory Synaptic Currents

Highlights

- Desensitized GABAAR can laterally diffuse between two adjacent GABAergic synapses
- Diffusion of desensitized GABAAR lowers IPSC amplitude at neighboring synapses
- GABAAR diffusion between synapses is modulated by calcium
- Moderate calcium influx induces GABAAR trapping at glutamatergic synapses

Authors

Emanuela de Luca, Tiziana Ravasenga, Enrica Maria Petrini, Alice Polenghi, Thierry Nieus, Stefania Guazzi, Andrea Barberis

Correspondence

andrea.barberis@iit.it

In Brief

de Luca et al. reveal that the lateral diffusion of postsynaptic GABAA receptor mediates an “information transfer” between adjacent dendritic inhibitory synapses. This mechanism represents a novel determinant shaping inhibitory synaptic transmission and is expected to fine-tune synaptic signaling.

de Luca et al., 2017, Neuron *95*, 63–69
July 5, 2017 © 2017 The Author(s). Published by Elsevier Inc.
http://dx.doi.org/10.1016/j.neuron.2017.06.022

CellPress

Inter-Synaptic Lateral Diffusion of GABAA Receptors Shapes Inhibitory Synaptic Currents

Emanuela de Luca,[1,3] Tiziana Ravasenga,[1,3] Enrica Maria Petrini,[1,3] Alice Polenghi,[1] Thierry Nieus,[1,2] Stefania Guazzi,[1] and Andrea Barberis[1,4,*]
[1]Neuroscience and Brain Technologies Department, Fondazione Istituto Italiano di Tecnologia, Via Morego 30, Genova, Italy
[2]Department of Biomedical and Clinical Sciences "L. Sacco," Università degli Studi di Milano, Via Grassi 74, 20157 Milan, Italy
[3]These authors contributed equally
[4]Lead Contact
*Correspondence: andrea.barberis@iit.it
http://dx.doi.org/10.1016/j.neuron.2017.06.022

SUMMARY

The lateral mobility of neurotransmitter receptors has been shown to tune synaptic signals. Here we report that GABAA receptors (GABAARs) can diffuse between adjacent dendritic GABAergic synapses in long-living desensitized states, thus laterally spreading "activation memories" between inhibitory synapses. Glutamatergic activity limits this inter-synaptic diffusion by trapping GABAARs at excitatory synapses. This novel form of activity-dependent hetero-synaptic interplay is likely to modulate dendritic synaptic signaling.

INTRODUCTION

The lateral diffusion of surface neurotransmitter receptor and its transient trapping at synapses is regulated by neuronal activity and plays a key role in adjusting receptor number at synapses during synaptic plasticity (Choquet and Triller, 2013). Moreover, the fast exchange between desensitized synaptic AMPA receptors and naive extrasynaptic receptors modulates the amplitude of glutamatergic synaptic currents (Constals et al., 2015; Heine et al., 2008). Receptor lateral mobility can therefore be an important determinant of synaptic transmission. To date, receptor diffusion has been examined only at the level of individual synapses, limiting our understanding of how diffusion shapes synaptic currents. It has never been investigated whether receptor lateral diffusion may transfer information between two or more adjacent synapses. We hypothesized that synaptic receptors in a given activation state at one synapse may diffuse and contact a neighboring synapse in the same conformational state, thus transmitting its activation history. In the present study, we tested this idea at inhibitory synapses, as the persistence of GABAA receptors (GABAARs) in long-living desensitized states (Overstreet et al., 2000; Petrini et al., 2011) may favor potential synaptic crosstalk mediated by the inter-synaptic diffusion of GABAARs. We report that following sustained stimulation of an individual GABAergic synapse, desensitized GABAARs laterally diffuse at neighboring dendritic GABAergic synapses, where they reduce the amplitude of inhibitory synaptic currents.

RESULTS

GABAA Receptors Diffuse between Synapses

To test whether GABAARs diffuse between two dendritic inhibitory synapses, we performed single particle tracking experiments (SPT) on endogenous GABAARs in cultured hippocampal neurons. The profile of the dendrites was identified by EGFP transfection and the position of inhibitory synapses by live immunostaining of vGAT (Figure 1A). During 1-min-long SPT experiments, we observed several α1-containing GABAARs contacting two adjacent inhibitory synapses (Figure 1B). Such inter-synaptic diffusion occurred in ~15% of synaptic GABAAR trajectories (n = 179, in 26 neurons from 8 cultures). The time required for GABAARs to contact two adjacent inhibitory synapses (typically 2–4 μm apart) ranged from a few hundred milliseconds to a few seconds (Figure 1C). Interestingly, the inter-synaptic displacement times were comparable to the kinetics of slow desensitized state(s) of GABAAR subtypes expressed at GABAergic synapses (Overstreet et al., 2000; Petrini et al., 2011). This result supports the hypothesis that receptors can swap between synapses in the desensitized state(s), thus mediating synaptic crosstalk. As reported in Figure 1D, the median diffusion coefficient (see STAR Methods) of GABAARs swapping among neighbor synapses was 0.07 $\mu m^2 s^{-1}$ (interquartile range [IQR] = 0.04 ÷ 0.13 $\mu m^2 s^{-1}$, n = 146). However, according to the free-boundary Brownian diffusion equation, such diffusion coefficient values accounted only for the slower inter-synaptic transition times, whereas the fastest events could not be fully predicted. Interestingly, we found that taking into account the narrow and elongated shape of dendrites, the diffusion coefficients in the longitudinal axis (see STAR Methods) were significantly higher than the transversal one (Figures S1A and S1B) and could explain inter-synaptic displacements even in the sub-second time range (Figure S1C). Model simulations based on the values of longitudinal diffusion coefficients (that are important for the inter-synaptic receptor diffusion) match the experimental distribution of inter-synaptic transition times (Figures S1D and S1E).

This is an open access article under the CC BY license (http://creativecommons.org/licenses/by/4.0/).

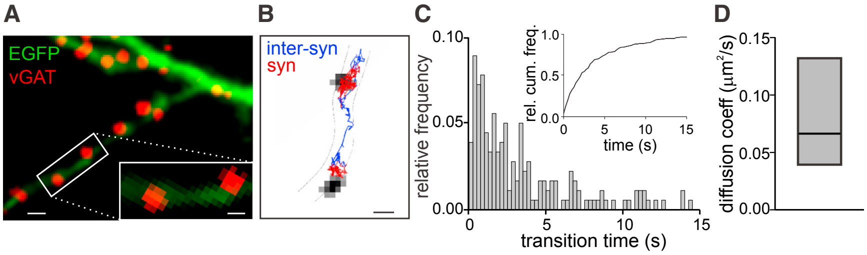

Figure 1. GABAARs Laterally Diffuse between Two Adjacent Inhibitory Synapses

(A) Representative live-stained inhibitory synapses (vGAT, red) in an EGFP-transfected neuron (green). Scale bar, 1 μm. Inset: magnification of the framed area. Scale bar, 500 nm.

(B) Reconstructed trajectory of α1-containing endogenous GABAAR diffusing at synaptic (red) and inter-synaptic (blue) compartments. Inhibitory synapses are in gray. Scale bar, 500 nm.

(C) Histogram and cumulative distribution (inset) of the inter-synaptic displacement time of endogenous GABAAR (n = 179, in 26 neurons from 8 cultures).

D) Diffusion coefficient of inter-synaptic GABAARs. Median = 0.066 $\mu m^2 s^{-1}$, IQR = 0.039 ÷ 0.132 $\mu m^2 s^{-1}$, n = 146, in 26 neurons from 8 cultures).

Intracellular Calcium Modulates GABAA Receptor Inter-synaptic Diffusion

Since the mobility of GABAARs depends on the intracellular calcium concentration (Bannai et al., 2009, 2015), we next studied how their inter-synaptic diffusion responds to the activation of the light-gated Ca^{2+}-permeable ionotropic glutamate receptor (LiGluK2) (Volgraf et al., 2006). LiGluK2 is an optogenetic tool to precisely control calcium influx at high spatial and temporal resolution (see STAR Methods, Figure S2A). Recombinant GluK2 receptors accumulate at glutamatergic synapses similarly to native ones (Martin et al., 2008), and so LiGluK2 activation is expected to mimic excitatory synaptic activity. In LiGluK2-GFP-transfected neurons, we used SPT to track the HA-tagged α1 subunit of GABAAR (HA-GABAAR), which is incorporated into GABAARs without affecting their surface expression, synaptic accumulation (Figure S2B), lateral mobility (Figure S2C), or inter-synaptic transitions (compare Figures 1C and 1D to Figures 2B and 2C). During LiGluK2 activation, HA-GABAARs were significantly less mobile in the inter-synaptic space (Figure 2A), as demonstrated by a lower inter-synaptic diffusion coefficient, rightward-shifted inter-synaptic transition time histogram and cumulative distribution, and increased time for inter-synaptic displacement (Figures 2B and 2C). Calcium entry therefore decreases the inter-synaptic mobility of HA-GABAARs.

To corroborate that GABAAR inter-synaptic diffusion depends on calcium, we exploited an altered form of LiGluK2, in which the substitution of glutamine 621 for an arginine in the pore lining M2 segment (Q/R editing) abolishes the GluK2 receptor's calcium permeability (Burnashev et al., 1995). As expected, LiGluK2(Q621R) displayed negligible calcium conductance (Figure S2A). In SPT experiments, the activation of LiGluK2(Q621R) did not significantly reduce the inter-synaptic diffusion coefficient of HA-tagged GABAAR (Figures 2D and 2E). However, LiGluK2(Q621R) activation did still cause a significant increase in GABAAR inter-synaptic displacement time (Figure 2F). A possible explanation for this partial effect might be that the depolarization induced by LiGluK2(Q621R) integrated over 1 min elicits a calcium increase through voltage-gated calcium channels (VGCCs). This hypothesis was tested by calcium imaging. Despite the Ca^{2+}-impermeability of LiGluK2(Q621R), its 1-min activation caused a mild intracellular calcium increase (30% of that mediated by wild-type [WT] LiGluK2) (Figure S2D). Importantly, such "residual" Ca^{2+} entry was completely prevented by the application of ω-conotoxin MVIIC (2 μm) and nifedipine (10 μm) (Figure S2D bottom). As a third method of testing calcium dependence, we next activated LiGluK2 receptors in the absence of extracellular calcium. Compared to control conditions, LiGluK2 activation in the absence of extracellular calcium did not significantly change either the inter-synaptic diffusion coefficient or the transition times of HA-α1 subunits (Figures 2G–2I). To further clarify the sources of Ca^{2+} responsible for the modulation of GABAAR inter-synaptic mobility, we next examined the role of VGCCs during LiGluK2 activation. The application of ω-conotoxin and nifedipine reduced the Ca^{2+} entry elicited by WT LiGluK2 by ~40%, as quantified in calcium imaging experiments (Figure S2D top), but left unchanged the LiGluK2-dependent effects on GABAAR inter-synaptic lateral mobility (Figures S2E and S2F). This indicates that upon LiGluK2 activation, direct Ca^{2+} entry through LiGluK2 (which accounts for ~60% of the total intracellular Ca^{2+} rise triggered by LiGluK2 opening) is the major player in the decrease of GABAAR diffusion between two adjacent synapses. Further control experiments ruled out any effect of the UV illumination on GABAAR lateral diffusion (Figure S2G) or on intracellular Ca^{2+} concentration (Figure S2H).

Activated Glutamatergic Synapses Interfere with GABAA Receptor Inter-synaptic Diffusion

As excitatory-like stimuli modulate inter-synaptic GABAAR diffusion, we considered a potential role for excitatory synapses in this phenomenon. We used the same experimental set-up to track inter-synaptic trajectories of HA-tagged GABAAR, but also analyzed glutamatergic synapses, identified by Homer1C-DsRed expression (Figure 3A). Interestingly, at glutamatergic synapses, LiGluK2 stimulation caused GABAARs to be markedly immobilized and confined (Figure 3B), indicated by the reduced synaptic diffusion coefficient, increased synaptic dwell time, and lower steady state of the mean square displacement (MSD) versus time curve (Figure 3C). These data reveal that the hetero-synaptic trapping of GABAARs at glutamatergic synapses plays a key role in the stimulus-dependent reduction of GABAAR inter-synaptic diffusion. In additional experiments, we found that at inhibitory synapses, LiGluK2 activation increases the mobilization of synaptic GABAARs, shown by their increased diffusion coefficient and decreased confinement (Figure S3). Taken together, our data show that light-controlled activation of glutamate receptors increases synaptic diffusion at inhibitory synapses and decreases inter-synaptic diffusion of GABAAR.

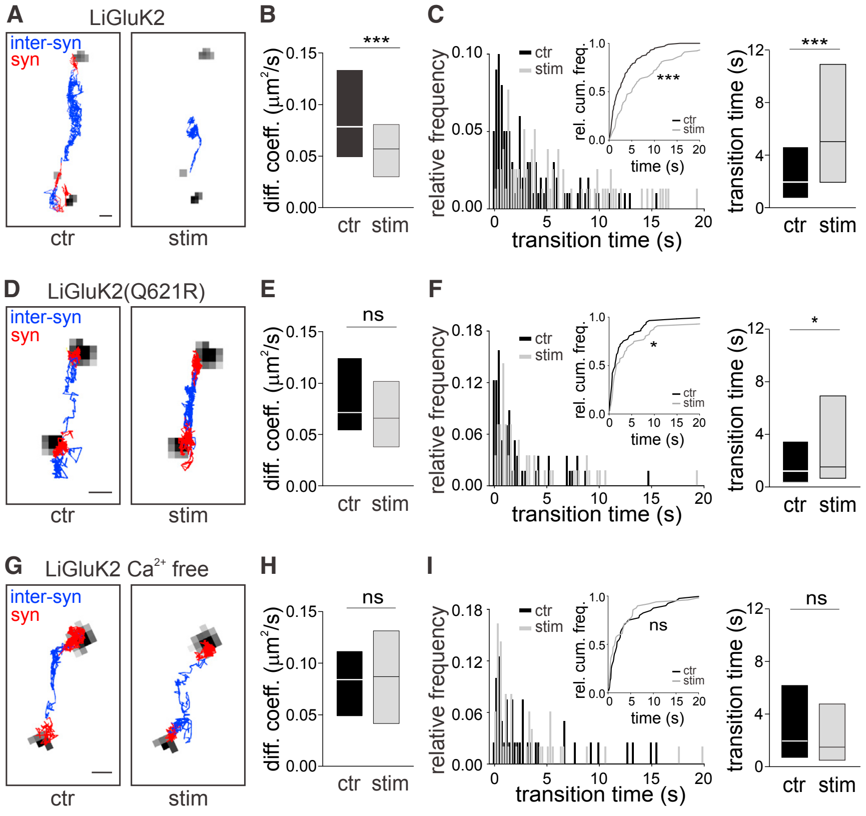

Figure 2. Modulation of GABAAR Inter-synaptic Mobility Is Ca^{2+} Dependent

(A) Reconstructed inter-synaptic (blue) and synaptic (red) trajectories of the same HA-GABAAR in the control (left) and upon LiGluK2 activation (right). Scale bar, 500 nm.

(B) Diffusion coefficient of inter-synaptic HA-GABAAR. Control (ctr): median = 0.078 $\mu m^2 s^{-1}$, IQR = 0.049–0.133 $\mu m^2 s^{-1}$, n = 55; LiGluK2 activation (stim): median = 0.057 $\mu m^2 s^{-1}$, IQR = 0.030–0.081 $\mu m^2 s^{-1}$, n = 44, $p < 0.001$, Mann-Whitney U-test; 37 neurons from 7 cultures.

(C) Left: Histogram and cumulative distribution of inter-synaptic displacement time in the control (black) and during LiGluK2 activation (gray). Right: HA-GABAAR inter-synaptic transition time. Ctr: median = 1.98 s, IQR = 0.75–4.64 s, n = 100; stim: median = 5.03 s, IQR = 1.95–10.87 s, n = 78, $p < 0.001$, Mann-Whitney U-test; 37 neurons from 7 cultures.

(D) Reconstructed trajectories of an individual HA-GABAAR in the control (left) and upon LiGluK2(Q621R) activation (right). Synaptic and inter-synaptic trajectories are represented in red and blue, respectively. Scale bar, 500 nm.

(E) Median diffusion coefficient of inter-synaptic HA-GABAAR in control (0.073 $\mu m^2 s^{-1}$, IQR = 0.055–0.126 $\mu m^2 s^{-1}$, n = 24) and LiGluK2(Q621R) activation (stim) (0.067 $\mu m^2 s^{-1}$, IQR = 0.038–0.103 $\mu m^2 s^{-1}$, n = 25), p = 0.332, Mann-Whitney U-test; 15 neurons from 3 cultures.

(F) Left: Histogram and cumulative distribution of inter-synaptic displacement times in the control (black) and during LiGluK2(Q621R) activation (gray). Right: HA-GABAAR inter-synaptic transition time. Ctr: median = 1.35 s, IQR = 0.50–3.63 s, n = 57; LiGluK2(Q621R) activation (stim): median = 1.68 s, IQR = 0.81–7.08 s, n = 56, $p < 0.05$, Mann-Whitney U-test; 15 neurons from 3 cultures.

(G) Reconstructed inter-synaptic (blue) and synaptic (red) HA-GABAAR trajectories in the control (left) and upon LiGluK2 activation in Ca^{2+}-free solution (right). Scale bar, 500 nm.

(H) Median diffusion coefficient of inter-synaptic HA-GABAAR in Ca^{2+} free solution. Control (ctr): 0.085 $\mu m^2 s^{-1}$, IQR = 0.049–0.112 $\mu m^2 s^{-1}$, n = 19; LiGluK2 activation (stim): 0.086 $\mu m^2 s^{-1}$, IQR = 0.040–0.130 $\mu m^2 s^{-1}$, n = 18, p = 0.939, Mann-Whitney U-test; 13 neurons from 5 cultures.

(I) Left: Histogram and cumulative distribution of inter-synaptic transition time in the control (black) and during LiGluK2 activation (gray) in Ca^{2+}-free solution. Right: HA-GABAAR inter-synaptic transition time. Ctr: median = 1.98 s, IQR = 0.73–6.21 s, n = 40; LiGluK2 activation in Ca^{2+}-free solution (stim): median = 1.5 s, IQR = 0.5–4.8 s, n = 49, p = 0.228, Mann-Whitney U-test; 13 neurons from 5 cultures. Data are represented as median ± IQR.

GABAA Receptor Inter-synaptic Diffusion Shapes Inhibitory Synaptic Current

Finally, we investigated the functional impact of inter-synaptic HA-tagged GABAAR diffusion on inhibitory synaptic transmission. We hypothesized that the spreading of GABAAR in long-living desensitized states could tune the availability of naive synaptic receptors at adjacent GABAergic synapses. To test this, we uncaged GABA (DPNI-GABA) at individual GABAergic synapses by UV-laser photolysis at diffraction limited spots (see STAR Methods and Figure S4A). With this approach, we compared the uncaging inhibitory postsynaptic currents (uIPSCs) recorded at a given GABAergic synapse (synapse A) before and after the induction of GABAAR desensitization by a uIPSC train delivered at a neighboring GABAergic synapse (synapse B) (Figure 4A). We observed that uIPSC trains (4 s, 16 Hz) transiently reduced uIPSC amplitude at synapses located 2–4 μm from the stimulated synapse (Figures 4B and 4C, black). When LiGluK2 receptors were activated (i.e., when GABAAR inter-synaptic diffusion was reduced), the extent of desensitization at neighboring synapses was significantly lower than in controls (Figures 4B and 4C, green). To further test the effect of receptor mobility on desensitization, we used the cross-link protocol (X-link) (Gerrow and Triller, 2014; Heine et al., 2008) to immobilize GABAARs (Figure 4D). Notably, when GABAAR was immobilized, uIPSC trains induced minimal desensitization at neighboring synapses (Figures 4B and 4C, red). These results were recapitulated when uIPSCs were mediated by endogenous GABAAR, ruling out possible artifacts due to α1-HA overexpression (Figure S4B). It may be posited that the steric hindrance of the X-link protocol interferes with neurotransmitter diffusion, which could account for the reduced GABAAR desensitization. To exclude this possibility, we X-linked neuroligin3 (NL3), a trans-membrane synaptic protein distinct from GABAAR. In these conditions, there was no significant reduction of synaptic desensitization (Figure S4C), demonstrating that the X-link protocol does not affect the diffusion of uncaged neurotransmitter. In order to demonstrate that the 4-s uIPSC train at 16 Hz effectively induced GABAAR desensitization, the amplitude of uIPSCs before and

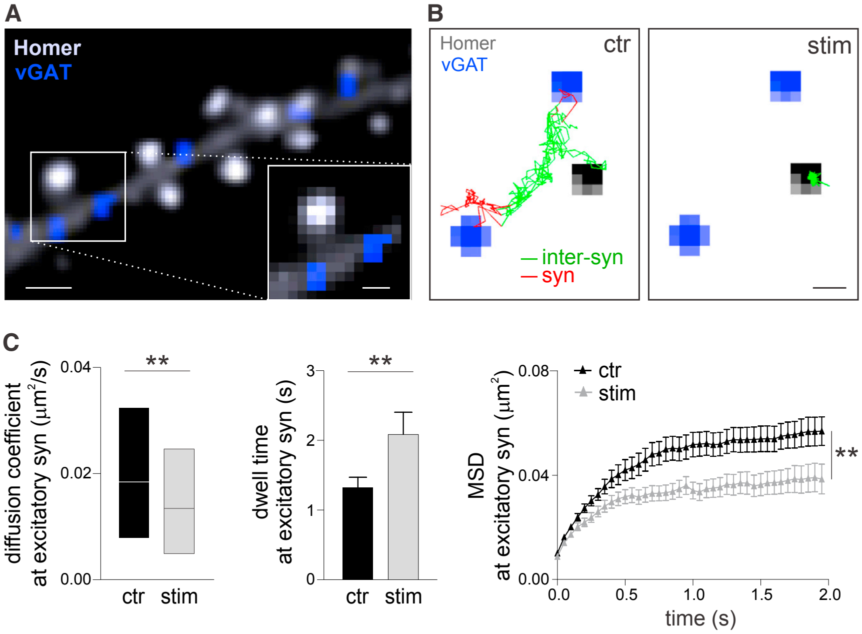

Figure 3. GABAAR Trapping at Glutamatergic Synapses upon LiGluK2 Activation Modulates GABAAR Inter-synaptic Diffusion

(A) Representative fluorescence image of inhibitory synapses (blue) and excitatory synapses (white). Scale bar, 1 μm. Inset: magnification of the framed area. Scale bar, 500 nm.

(B) Reconstructed inter-synaptic HA-GABAAR trajectories in the control (left) and upon LiGluK2 activation (right). Inhibitory synapses are in blue and excitatory synapses in gray. Inhibitory synaptic and inter-synaptic trajectories are represented in red and green, respectively. Scale bar, 500 nm.

(C) Left: Diffusion coefficient of HA-GABAAR at excitatory synapses in the control (ctr) (median = 0.017 $\mu m^2 s^{-1}$, IQR = 0.006–0.032 $\mu m^2 s^{-1}$, n = 187) and upon LiGluK2 activation (stim) (median = 0.012 $\mu m^2 s^{-1}$, IQR = 0.004–0.023 $\mu m^2 s^{-1}$, n = 208), $p < 0.01$, Mann-Whitney U-test; 15 neurons from 3 cultures. Middle: Dwell time of HA-GABAAR at excitatory synapses. Control (ctr), 1.3 ± 0.1 s, n = 122; LiGluK2 activation (stim), 2.1 ± 0.3 s, n = 100, $p < 0.01$, Mann-Whitney U-test; 15 neurons from 3 cultures. Right: MSD versus time plot of HA-GABAARs at excitatory synapses (steady state: ctr = 0.056 ± 0.005 μm^2, n = 79; stim = 0.039 ± 0.005 μm^2, n = 90, $p < 0.01$, Student's t test); 15 neurons from 3 cultures. Unless otherwise stated, data are represented as mean ± SEM. Boxplots indicate the median and IQR.

after the uIPSC train was tested at the same synapse at which the train was delivered. We found that 660 ms after the uIPSC train, uIPSC amplitude was depressed by ~60%, thus indicating massive desensitization (Figure S4D). In X-link conditions, the extent of desensitization was further increased, with uIPSC amplitude 660 ms after the train reduced by ~73% (Figure S4D). These results show that the exchange of synaptic receptors with naive ones by lateral diffusion reduces the amount of synaptic desensitization, in line with what is observed for AMPA receptors at glutamatergic synapses (Heine et al., 2008). Interestingly, the X-link increased the desensitization "at the same synapses" due to the trapping of desensitized receptors, while it reduced the desensitization at "neighboring synapses," due to the inability of desensitized receptors to spread from the desensitized synapse. We next assessed whether receptors in the desensitized state are able to diffuse between two synapses. To this end, we studied the inter-synaptic displacements of HA-GABAAR through SPT experiments during the bath application of 100 μM GABA to induce massive GABAAR desensitization. The HA-GABAAR diffusion coefficient and inter-synaptic transition times were comparable in control conditions and in 100 μM GABA (Figures S4E and S4F), thus confirming that desensitized GABAARs are able to diffuse inter-synaptically. Since receptor saturation at the stimulated synapse would maximize the number of desensitized receptors leaving for neighboring synapses, we also examined synaptic GABAAR saturation by quantifying uIPSC variability. The coefficient of variation (CV) of uIPSCs elicited by laser pulses delivered at the same synapse every 10 s to uncage 1 mM and 2 mM GABA were similar, indicating that saturation was achieved. Hence, individual pulses were sufficient to saturate postsynaptic receptors (Figure S4G). Moreover, the comparable CV of uIPSCs mediated by native, HA-tagged, and X-linked GABAARs (Figure S4G) suggests that GABA uncaging pulses are saturating in all of the conditions tested in the present study. Taken together, these experiments indicate that the diffusion of desensitized GABAARs between adjacent dendritic GABAergic synapses shapes inhibitory synaptic currents (Figure 4E).

DISCUSSION

The present study provides evidence for a novel mechanism of synaptic crosstalk based on the diffusion of desensitized GABAARs between inhibitory synapses (Figure 4E). We demonstrate that a given inhibitory synapse may transfer the "memory" of its recent activation to neighboring inhibitory synapses. Receptor lateral mobility is a fundamental determinant of synaptic function at glutamatergic synapses, where AMPA receptor diffusion between synaptic and extrasynaptic areas modulates the amplitude of synaptic excitatory currents (Constals et al., 2015; Heine et al., 2008). The present study shows for the first time that surface receptor diffusion can functionally connect two distinct synapses. At inhibitory synapses, this new form of inter-synaptic crosstalk relies on the fine temporal relationship between GABAAR gating kinetics and the time needed for GABAAR to undergo inter-synaptic displacements. Following prolonged activation, GABAARs are absorbed into long-living desensitized states lasting up to tens of seconds (Overstreet et al., 2000; Petrini et al., 2011). This timing is a distinctive GABAAR gating feature that represents a key requisite for efficient inter-synaptic communication. In this context, it should be noted that the time course of current amplitude reduction at neighboring synapses, here shown to depend on inter-synaptic receptor swap, is also influenced by the rate of GABAAR exit

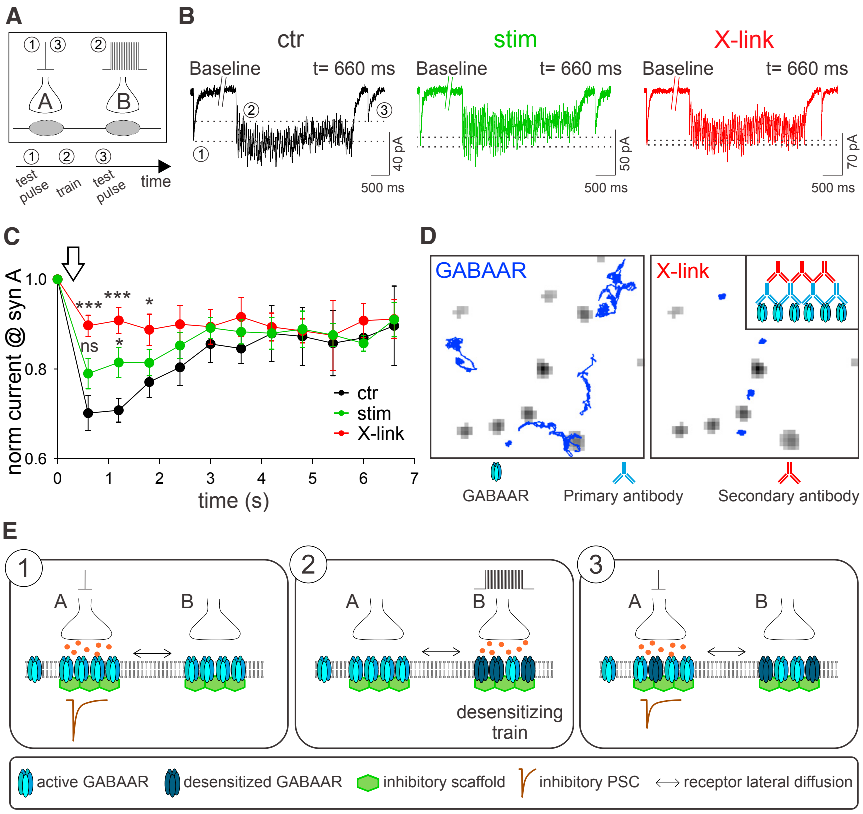

Figure 4. Inter-synaptic GABAAR Diffusion Shapes Inhibitory Synaptic Currents

(A) Schematic of the GABA uncaging protocol. (1) A single UV laser pulse (0.5 ms, 0.1 mW) is applied at "synapse A" to record a baseline uIPSC. (2) A UV laser train pulse (4 s at 16 Hz) induces synaptic GABAAR desensitization at "synapse B." (3) A UV laser pulse at "synapse A" monitors the modulation of uIPSC after the desensitizing train at "synapse B."

(B) Representative traces of uncaging currents recorded in the control (black), during LiGluK2 activation (green), and upon the X-link protocol (red), before (1) and 660 ms after (3) the UV laser train pulse, as described in (A).

(C) Normalized recovery of uIPSC amplitude induced at "synapse A" after the delivery of the UV laser train pulse at "synapse B" (arrow) in the control (black), LiGluK2 (green), and X-link (red). At 660 ms: ctr = 0.71 ± 0.03, n = 12; 11 neurons in 5 cultures; stim = 0.82 ± 0.04, n = 12; 6 neurons in 4 cultures; X-link = 0.91 ± 0.03, n = 14; 9 neurons in 4 cultures; $p < 0.05$ ctr versus stim, $p < 0.001$ ctr versus X-link, Mann-Whitney U-test. Data are represented as means ± SEM.

(D) Reconstructed trajectories of GABAARs (blue) in control (left) and upon GABAAR X-link (right). Inhibitory synapses are in gray. Inset: schematic of the X-link protocol.

(E) Model for the modulation of uIPSCs by inter-synaptic lateral diffusion of desensitized GABAARs. Upon sustained inhibitory synaptic activity, the amplitude of synaptic responses at individual synapses is reduced by intruder desensitized GABAARs from neighboring synapses. Impeding GABAAR lateral diffusion prevents such modulation of inhibitory synaptic transmission.

from desensitization, which in turn depends on the duration/concentration of GABA pulses (Petrini et al., 2011).

Another important determinant for the efficiency of inter-synaptic crosstalk is the level of synaptic receptor saturation during synaptic activity. Indeed, in a saturation regime, when all the postsynaptic receptors are activated, any decrease in the number of "activatable" receptors would result in a sizable reduction of synaptic current amplitude. In our experiments, a single GABA uncaging pulse saturates postsynaptic receptors in all conditions tested (Figure S4G), while a number of studies have shown that at central synapses, uniquantal neurotransmitter release may either be saturating or sub-saturating (Auger and Marty, 1997; Poncer et al., 1996). Nevertheless, saturation of postsynaptic receptor clusters can be still attained by (1) repetitive synaptic activations and/or (2) multi-vesicular release (Rudolph et al., 2015). Thus, the mechanisms proposed here may also occur during sustained synaptic activity and/or specific releasing patterns at synapses that would not be saturated by a single neurotransmitter vesicle.

In the present study, we investigated the role of calcium in the GABAAR inter-synaptic mobility by exploiting LiGluK2, an optogenetic tool that allows the control of Ca^{2+} inflow with high temporal precision. We observed that intracellular Ca^{2+} rise mediated by LiGluK2 activation reduces GABAAR mobility, thus limiting inter-synaptic crosstalk. In contrast, a previous study found that sustained network stimulation promoted GABAAR mobilization (Bannai et al., 2009). The reason for this discrepancy is most likely due to the different type of stimulation used. While Bannai et al. (2009) induced massive Ca^{2+} entry by enhancing excitation or by blocking inhibition, in our experiments we elicited a mild and controlled Ca^{2+} rise, likely mimicking glutamatergic synaptic activity. Consistent with this explanation, it has been recently demonstrated that sustained Ca^{2+} entry increases GABAAR mobility, whereas moderate Ca^{2+} elevation induces GABAAR immobilization (Bannai et al., 2015). In this scenario, glutamatergic synapses tightly intercalated with dendritic GABAergic synapses (i.e., the typical synapse distribution at proximal dendrites of pyramidal neurons; Megías et al., 2001) are optimally located to tune GABAAR inter-synaptic diffusion through mild and localized dendritic Ca^{2+} inflow. However, such modulation of GABAAR diffusion by Ca^{2+} may be significantly different in specific neuronal sub-compartments, such as the somata of pyramidal neurons that exclusively receive inhibitory inputs (Klausberger and Somogyi, 2008), or the distal dendrites of pyramidal neurons, which mainly have glutamatergic synapses (Megías et al., 2001). In addition, the impact of Ca^{2+} dynamics on GABAAR diffusion in micro- and nano-domains is still to be elucidated and is expected to add further complexity to the lateral-diffusion-mediated synaptic crosstalk.

An important finding of this study is that in response to glutamatergic stimulation (mimicked by LiGluK2 activation), GABAARs are trapped at glutamatergic synapses, which significantly limits GABAAR inter-synaptic diffusion. Although the presence of GABAARs at excitatory synapses has already been described (Nusser et al., 1996; Renner et al., 2012), this is the first evidence that a hetero-synaptic interaction is modulated by activity. It has been argued previously that in steady-state conditions, local molecular crowding at the glutamatergic postsynaptic density (PSD) may reduce the GABAAR diffusion coefficient without any increase in either receptor accumulation or dwell time at excitatory synapses (Renner et al., 2012). According to this hypothesis, the increased GABAAR dwell time at glutamatergic synapses reported here would suggest molecular interactions between GABAAR and the glutamatergic synaptic scaffold. Nevertheless, Renner et al. (2012) also reported that the molecular crowding can induce transient accumulation of GABAARs at glutamatergic synapses. In this alternative scenario, Ca^{2+}-dependent rearrangements of the excitatory PSD (Opazo et al., 2010) may temporarily trap GABAARs at excitatory synapses, thus explaining our observation without invoking the binding of GABAAR at the glutamatergic PSD.

It might be suggested that uncaged GABA could directly activate and desensitize GABAARs at nearby synapses due to synapse-to-synapse spillover. This possibility can be ruled out by the negligible desensitization observed at neighboring synapses following uIPSC trains when GABAARs are immobilized by X-link (Figure 4 and Figure S4B), a procedure that does not prevent GABA spillover (Figure S4C). However, it is likely that uncaged GABA diffusing outside the synapse reaches and desensitizes peri-and extra-synaptic GABAARs, resulting in their contribution to the inter-synaptic crosstalk. Indeed, neurotransmitter diffusion in the extrasynaptic space is a feature of synaptic transmission, especially under repetitive synaptic activation (Rudolph et al., 2015). Hence, during sustained activity at a given synapse that elicits sizable agonist spillover in the extrasynaptic space, a larger population of desensitized GABAARs (including peri- and extrasynaptic receptors) would modulate the efficacy of neighboring synapses by lateral diffusion. Therefore, it can be speculated that the inclusion of desensitized peri- or extrasynaptic GABAARs at neighboring synapses during sustained inhibitory synaptic activity would be an extended feature of synaptic crosstalk through GABAAR lateral diffusion.

Conventional synaptic transmission assumes that synapses work independently, a condition that maximizes information storage in the brain (Barbour, 2001). However, several lines of evidence challenge this view, favoring the idea that under specific conditions, the activation of a given synapse may influence the function of surrounding synapses by diffusion-driven events such as neurotransmitter spillover (Rudolph et al., 2015) or local changes of the ionic driving force (Doyon et al., 2011). The present study identifies a novel additional mechanism of inter-synaptic information transfer, finding that glutamatergic synaptic activity may switch the behavior of inhibitory synapses from "crosstalking" to "working independently," with important implications for dendritic synaptic signaling.

STAR★METHODS

Detailed methods are provided in the online version of this paper and include the following:

- KEY RESOURCES TABLE
- CONTACT FOR REAGENT AND RESOURCE SHARING
- EXPERIMENTAL MODEL AND SUBJECT DETAILS
  - Primary neuronal cultures
- METHOD DETAILS
  - Plasmid constructs
  - Transfection and synapse identification
  - Single particle tracking
  - Calcium imaging and pharmacology
  - Immunocytochemistry
  - Electrophysiology and GABA uncaging
  - Cross-link (X-link) protocol
  - Light-Activated glutamate receptors (LiGluK2)
- QUANTIFICATION AND STATISTICAL ANALYSIS
- DATA AND SOFTWARE AVAILABILITY

SUPPLEMENTAL INFORMATION

Supplemental Information includes four figures and can be found with this article online at http://dx.doi.org/10.1016/j.neuron.2017.06.022.

AUTHOR CONTRIBUTIONS

Conceptualization, A.B.; Methodology, A.B., E.M.P., T.R., T.N., E.d.L., and A.P.; Validation, A.B. and E.M.P.; Formal Analysis, E.d.L., T.R., E.M.P., A.P., T.N., and A.B.; Investigation, E.d.L., T.R., E.M.P., A.P., and T.N.; Resources, S.G.; Writing – original Draft, A.B.; Writing – Review & Editing, A.B. and E.M.P.; Visualization, E.M.P., E.d.L., A.P., and T.N.; Supervision, A.B.; Project Administration, A.B.; Funding Acquisition, A.B.

ACKNOWLEDGMENTS

We are grateful to E. Isacoff, D. Choquet, and P. Scheiffele for kindly providing the LiGluK2-GFP, Homer-DsRed, and NL3-HA constructs, respectively. We thank D. Choquet and L. Cognet for some SPT analysis software. We thank P. Gorostiza and D. Trauner for providing us with MAG. We thank G. Aletti for previous discussion on the mathematics underlying the Brownian motion. This work has been supported by Telethon-Italy (GGP11043) and Compagnia di San Paolo (ROL-4318).

Received: March 31, 2017
Revised: May 15, 2017
Accepted: June 14, 2017
Published: July 5, 2017

REFERENCES

Auger, C., and Marty, A. (1997). Heterogeneity of functional synaptic parameters among single release sites. Neuron *19*, 139–150.

Bannai, H., Lévi, S., Schweizer, C., Inoue, T., Launey, T., Racine, V., Sibarita, J.B., Mikoshiba, K., and Triller, A. (2009). Activity-dependent tuning of inhibitory neurotransmission based on GABAAR diffusion dynamics. Neuron *62*, 670–682.

Bannai, H., Niwa, F., Sherwood, M.W., Shrivastava, A.N., Arizono, M., Miyamoto, A., Sugiura, K., Lévi, S., Triller, A., and Mikoshiba, K. (2015). Bidirectional Control of Synaptic GABAAR Clustering by Glutamate and Calcium. Cell Rep. *13*, 2768–2780.

Barbour, B. (2001). An evaluation of synapse independence. J. Neurosci. *21*, 7969–7984.

Budreck, E.C., and Scheiffele, P. (2007). Neuroligin-3 is a neuronal adhesion protein at GABAergic and glutamatergic synapses. Eur. J. Neurosci. *26*, 1738–1748.

Burnashev, N., Zhou, Z., Neher, E., and Sakmann, B. (1995). Fractional calcium currents through recombinant GluR channels of the NMDA, AMPA and kainate receptor subtypes. J. Physiol. *485*, 403–418.

Choquet, D., and Triller, A. (2013). The dynamic synapse. Neuron *80*, 691–703.

Constals, A., Penn, A.C., Compans, B., Toulmé, E., Phillipat, A., Marais, S., Retailleau, N., Hafner, A.S., Coussen, F., Hosy, E., and Choquet, D. (2015). Glutamate-induced AMPA receptor desensitization increases their mobility and modulates short-term plasticity through unbinding from Stargazin. Neuron *85*, 787–803.

Doyon, N., Prescott, S.A., Castonguay, A., Godin, A.G., Kröger, H., and De Koninck, Y. (2011). Efficacy of synaptic inhibition depends on multiple, dynamically interacting mechanisms implicated in chloride homeostasis. PLoS Comput. Biol. *7*, e1002149.

Gerrow, K., and Triller, A. (2014). GABAA receptor subunit composition and competition at synapses are tuned by GABAB receptor activity. Mol. Cell. Neurosci. *60*, 97–107.

Heine, M., Groc, L., Frischknecht, R., Béïque, J.C., Lounis, B., Rumbaugh, G., Huganir, R.L., Cognet, L., and Choquet, D. (2008). Surface mobility of postsynaptic AMPARs tunes synaptic transmission. Science *320*, 201–205.

Klausberger, T., and Somogyi, P. (2008). Neuronal diversity and temporal dynamics: the unity of hippocampal circuit operations. Science *321*, 53–57.

Martin, S., Bouschet, T., Jenkins, E.L., Nishimune, A., and Henley, J.M. (2008). Bidirectional regulation of kainate receptor surface expression in hippocampal neurons. J. Biol. Chem. *283*, 36435–36440.

Megías, M., Emri, Z., Freund, T.F., and Gulyás, A.I. (2001). Total number and distribution of inhibitory and excitatory synapses on hippocampal CA1 pyramidal cells. Neuroscience *102*, 527–540.

Nusser, Z., Sieghart, W., Stephenson, F.A., and Somogyi, P. (1996). The alpha 6 subunit of the GABAA receptor is concentrated in both inhibitory and excitatory synapses on cerebellar granule cells. J. Neurosci. *16*, 103–114.

Opazo, P., Labrecque, S., Tigaret, C.M., Frouin, A., Wiseman, P.W., De Koninck, P., and Choquet, D. (2010). CaMKII triggers the diffusional trapping of surface AMPARs through phosphorylation of stargazin. Neuron *67*, 239–252.

Overstreet, L.S., Jones, M.V., and Westbrook, G.L. (2000). Slow desensitization regulates the availability of synaptic GABA(A) receptors. J. Neurosci. *20*, 7914–7921.

Petrini, E.M., Nieus, T., Ravasenga, T., Succol, F., Guazzi, S., Benfenati, F., and Barberis, A. (2011). Influence of GABAAR monoliganded states on GABAergic responses. J. Neurosci. *31*, 1752–1761.

Petrini, E.M., Ravasenga, T., Hausrat, T.J., Iurilli, G., Olcese, U., Racine, V., Sibarita, J.B., Jacob, T.C., Moss, S.J., Benfenati, F., et al. (2014). Synaptic recruitment of gephyrin regulates surface GABAA receptor dynamics for the expression of inhibitory LTP. Nat. Commun. *5*, 3921.

Poncer, J.C., Dürr, R., Gähwiler, B.H., and Thompson, S.M. (1996). Modulation of synaptic GABAA receptor function by benzodiazepines in area CA3 of rat hippocampal slice cultures. Neuropharmacology *35*, 1169–1179.

Racine, V., Hertzog, A., Jouanneau, J., Salamero, J., Kervrann, C., and Sibarita, J.-B. (2006). Multiple-target tracking of 3D fluorescent objects based on simulated annealing. In 3rd IEEE Int Symp Biomed Imaging 1-3, pp. 1020–1023.

Renner, M., Schweizer, C., Bannai, H., Triller, A., and Lévi, S. (2012). Diffusion barriers constrain receptors at synapses. PLoS ONE *7*, e43032.

Rudolph, S., Tsai, M.C., von Gersdorff, H., and Wadiche, J.I. (2015). The ubiquitous nature of multivesicular release. Trends Neurosci. *38*, 428–438.

Trigo, F.F., Papageorgiou, G., Corrie, J.E., and Ogden, D. (2009). Laser photolysis of DPNI-GABA, a tool for investigating the properties and distribution of GABA receptors and for silencing neurons in situ. J. Neurosci. Methods *181*, 159–169.

Volgraf, M., Gorostiza, P., Numano, R., Kramer, R.H., Isacoff, E.Y., and Trauner, D. (2006). Allosteric control of an ionotropic glutamate receptor with an optical switch. Nat. Chem. Biol. *2*, 47–52.

STAR★METHODS

KEY RESOURCES TABLE

REAGENT or RESOURCE	SOURCE	IDENTIFIER
Antibodies		
Rabbit polyclonal anti-GABAARα1	Alomone	AGA-001, RRID: AB_2039862
Rabbit polyclonal anti-GABAARγ2	Alomone	AGA-005; RRID: AB_2039870
Rat monoclonal anti-HA	Roche	1186742300, RRID: AB_10094468
Anti vGAT-oyster 550	SynapticSystems	131103C3, RRID: AB_887867
Anti vGAT-oyster 650	SynapticSystems	131103C5, RRID: AB_2254821
Mouse anti-vGAT	SynapticSystems	131011, RRID: AB_887872
QDot 655 goat F(ab')2 anti mouse IgG	Thermo Fisher	Q11022MP, RRID: Q11022MP
QDot 625 goat F(ab')2 anti mouse IgG	Thermo Fisher	A10195, RRID: AB_2534020
QDot 655 goat F(ab')2 anti rabbit IgG	Thermo Fisher	Q11422MP, RRID: AB_10375438
Chemicals, Peptides, and Recombinant Proteins		
GABA	Sigma	A2129
ω-conotoxin MVIIC	Tocris Bioscience	1084/100U
nifedipine	Sigma	N-7634
Rhod-2	Thermo Fisher	R 14220
DAKO fluorescent mounting medium	DAKO	S302380-2
DPNI-GABA	Tocris Bioscience	2991-10
MAG	Gift from Trauner D. and Gorostiza P.	N/A
Casein	Vector lab	SP 5020
Critical Commercial Assays		
Effectene	QIAGEN	301427
QuickChange II Site-Directed Mutagenesis Kit	Agilent Technologies	200524
Experimental Models: Organisms/Strains		
Mouse: Wild-type (C57BL/6J)	Harlan	C57BL/6JRccHsd
Recombinant DNA		
pEGFP-N1	Clontech	Cat# 632162
pCDM8-α1-HA	This paper	N/A
pcDNA3.1 HA-Nlgn3 (NL3)	Budreck and Scheiffele, 2007	N/A
pTR-hSyn Grik2-L439C (LiGluK2)-GFP	Volgraf et al., 2006	N/A
LiGluK2-GFP-Q621R	This paper	N/A
Homer1c-DsRed	Opazo et al., 2010	N/A
Software and Algorithms		
Metamorph 7.8	Molecular Devices; RRID:SCR_002368	https://www.moleculardevices.com/systems/metamorphresearch-imaging/metamorph-microscopy-automationand-image-analysis-software; RRID: SCR_002368
Clampex 10.2	Molecular Devices	https://www.moleculardevices.com/systems/conventional-patch-clamp/pclamp-10-software; RRID: BDSC_14352
Clampfit 10	Molecular Devices	https://www.moleculardevices.com/systems/conventional-patch-clamp/pclamp-10-software; RRID: BDSC_14352
MATLAB	Mathworks	http://www.mathworks.com; RRID: SCR_001622
GraphPad Prism 5	GraphPad	https://www.graphpad.com/scientific-software/prism/; RRID: SCR_002798

(*Continued on next page*)

Continued

REAGENT or RESOURCE	SOURCE	IDENTIFIER
KyPlot 5.0	KyensLab	http://kyenslab-inc.software.informer.com/
Python Language Reference 2. 7	Python Software Foundation	https://www.python.org; RRID: SCR_008394
Custom program written for MATLAB to reconnect QD trajectories	Petrini et al., 2014; from D Choquet and L Cognet	N/A
Custom program for SPT quantifications, based on MSD fit	Petrini et al., 2014; from D Choquet and A Serge	N/A
Custom Python script to quantify diffusion coefficients based on the Gaussian fit	This paper	N/A
Custom Python script to simulate inter-synaptic transitions	This paper	N/A

CONTACT FOR REAGENT AND RESOURCE SHARING

Further information and requests for resources and reagents should be directed to and will be fulfilled by the Lead Contact, Andrea Barberis (andrea.barberis@iit.it).

EXPERIMENTAL MODEL AND SUBJECT DETAILS

Primary neuronal cultures

All the experiments were carried out in accordance with the guidelines established by the European Community Council and were approved by the Italian Ministry of Health. Primary cultures of hippocampal neurons were prepared from P0-P1 C57BL/6J mice of either sex. Neurons were plated at a density of 60×10^3 cells/cm^2 on poly-D-lysine pre-coated glass coverslips and kept in serum-free Neurobasal-A medium (Thermo Fisher, Italy) supplemented with Glutamax (Invitrogen, Italy) 1%, B-27 (Invitrogen, Italy) 2% and Gentamycin 5 mg/ml at 37°C in 5% CO2. All the experiments were performed at 12-16 Days in Vitro (DIV).

METHOD DETAILS

Plasmid constructs

EGFP was encoded by the pEGFP-N1 plasmid (Clontech, Italy). Hemagglutinin (HA)-tagged α1 GABAA receptor protein was obtained by introducing an oligonucleotide encoding for HA between the IV and V aminoacid of the mature protein in the pCDM8-α1subunit GABAAR plasmid, taking advantage of the Agilent mutagenesis kit. HA-tagged NL3 plasmid (kindly provided by P. Scheiffele) contains the HA sequence at the 5′ of the mature NL3 protein (Budreck and Scheiffele, 2007). The Homer-DsRed plasmid (kindly provided by D. Choquet) encodes for DsRed at the N terminus of Homer-1C (Opazo et al., 2010). LiGluK2-GFP was kindly provided by E. Isacoff (Volgraf et al., 2006). LiGluK2(Q621R) was generated by site directed mutagenesis. All constructs were verified by DNA sequencing.

Transfection and synapse identification

Neurons were transfected at DIV 6-7 using the Effectene kit (QIAGEN, Germany) following the protocol proposed by the company. Hippocampal neurons for single particle tracking (SPT) experiments in basal conditions were co-transfected with pEGFP-N1 to delineate the profile of the dendrites along with the plasmid encoding for the HA-tagged α1 subunit of GABAAR. GABAergic synapses were identified by live immunostaining of v-GAT, by incubating neurons for 30 min at 37°C with either the anti-vGAT-Oyster550 or the anti-vGAT-Oyster650 antibodies (Synaptic Systems, Germany) diluted in Neurobasal-A medium. In the SPT and electrophysiology experiments involving the activation of light-gated glutamate receptors (LiGluK2), hippocampal neurons were co-transfected with plasmids encoding for LiGluK2-GFP or LiGluK2(Q621R)-GFP along with the plasmid encoding for the HA-tagged α1 subunit of GABAAR. Synapses were identified as detailed above with the anti-vGAT-Oyster550 antibody. For the experiments focusing on GABAAR diffusion at glutamatergic synapses before and after LiGluK2 activation, hippocampal neurons were triple transfected with plasmid encoding for LiGluK2-GFP, HA-tagged α1 subunit of GABAAR and Homer1c-DsRed (to identify glutamatergic synapses); inhibitory synapses were live labeled with anti-vGAT-Oyster650 antibodies. In the NL3 X-link experiments (Figure S4C) hippocampal neurons coexpressed LiGluK2-GFP and NL3-HA plasmids. Inhibitory synapses were identified with the anti-vGAT-Oyster550 antibody.

Single particle tracking

Imaging

Quantum Dot (QD) staining of surface GABAAR (or NLG3) was performed as previously described (Petrini et al., 2014). Briefly, rabbit anti-α1 (Alomone, Israel) or mouse anti-HA antibody (Roche, Italy) were premixed with anti-rabbit QD 655, anti-mouse

QD 655 or anti-mouse 625 (Invitrogen, Italy) for 30 min in the presence of casein (Vector lab, Italy) to prevent non-specific binding. Neurons were then incubated with the diluted antibody-QD premix for 3 min at room temperature. SPT experiments were performed using an inverted microscope (Eclipse Ti, Nikon, Japan) equipped with a 100X oil, 1.4 NA immersion objective and a back-illuminated EMCCD camera Photometric Quantem 512S (pixel size, 160 nm). Samples were illuminated by exploiting a diode-based illumination device (Lumencor, SpectraX Light Engine, Optoprim, Italy). QD fluorescence was monitored over time by acquiring 1200 consecutive frames at 20 Hz using the Metamorph software (ver. 7.8, Molecular Devices, USA). Inhibitory synapses were identified as detailed above by immunolabeling vGAT-Oyster 550 or 650 antibodies (depending on the experiment). During the experiments, neurons were kept at 32°C (TC-324B Warner Instrument Corporation, CT, USA) in an open chamber and continuously superfused with the recording solution (see below) at the rate of 12 mL/hr. In the experiments aimed at probing the lateral diffusion of desensitized receptors (Figures S4E and S4F), SPT recordings in control and in the presence of GABA were performed on the same neuron before and after replacing the control recording solution with that supplemented with GABA 100 μM, in order to ensure rapid and controlled solution exchange. The SPT experiments involving LiGluK2 activation were performed as follows: i) ctr (LiGluK2 closed): 1200 consecutive frames at 20 Hz, with 490 nm light illumination; ii) stim (LiGluK2 open): 1200 consecutive frames at 20 Hz illuminating with the UV light at 380 nm. Taking advantage of their wide excitation spectrum, QD could be imaged at both excitation wavelengths. When VGCC blockers were used, neurons were incubated with ω-conotoxin MVIIC (2 μM) and nifedipine (10 μM) for 8 min before SPT recordings, a period sufficient to achieve the complete block of P/Q-, N- and L-type VGCCs (Figure S2E).

Analysis

Single QDs, recognized by their diffraction-limited fluorescence spot shape and characteristic blinking were tracked with 50 ms time resolution. QD spatial coordinates were identified in each frame as sets of > 4 connected pixels using two dimensional object wavelet-based localization at sub-diffraction limited resolution (~40 nm) with MIA software based on simulated annealing algorithm (Racine et al., 2006). Continuous tracking between blinks was performed with an implemented version of custom made software originally written in MATLAB (Mathworks, Italy) in Dr Choquet's lab. This method is based on a QD maximal allowable displacement (4 pixels) during a maximal allowable duration of the dark period (25 frames, corresponding to 1.25 s acquisition). This stringent reconnection of trajectories across QD blinking combined with the highly diluted QD labeling have been set to avoid erroneous reconnection of neighboring QD in the same trajectory and to provide unambiguous observations of individual receptor QD complex trajectories. Please note that the trajectories in the Figures have been reconnected throughout QD blinking events. Receptor trajectories were defined as "synaptic" (or "extrasynaptic") when their spatial coordinates coincided (or not) with those of the localization of the postsynaptic compartment. Since inhibitory synapses were identified by presynaptic vGAT labeling, postsynaptic compartments were defined as a 2-pixel enlargement of vGAT staining. Although the definition of the compartments was diffraction limited, the sub-wavelength resolution of the single particle detection (~40 nm) allowed accurate description of receptor mobility within such small regions. Instantaneous diffusion coefficients, D, were calculated from linear fits of the n = 1–4 values of the MSD versus time plot, according to the equation:

$$\langle r^2 \rangle = \left[\sum_{i=1}^{(N-n)} (X_{i+n} - X_i)^2 + (Y_{i+n} - Y_i)^2 \Big/ (N-n) \right] dt \qquad \text{(Equation 1)}$$

The diffusive properties of the mobile receptor population were described as their median ± interquartile range (IQR), defined as the interval between the 25th and 75th percentiles. The analysis was blindly performed.

Analysis and model simulations in a bounded diffusion space

In order to obtain independent estimates of the diffusion coefficient with respect to the standard procedure of fitting the first points of the MSD versus time curve, we designed an alternative approach and generated a new custom code. The new script was implemented in Python to quantify diffusion coefficients based on the Gaussian fit.

In a standard Brownian motion the 2D x, y coordinates of a receptor can be computed as:

$$\begin{cases} x_t = \sqrt{D_x\, dt}\, \varphi + \mu_x\, dt \\ y_t = \sqrt{D_y\, dt}\, \varphi + \mu_y\, dt \end{cases} \qquad \text{(Equation 2)}$$

where x_t and y_t are the coordinates at time t, $D_{x,y,}$ the diffusion coefficients, $\mu_{x,y}$ the drift coefficients, φ a Gaussian noise (mean 0, standard deviation 1) and dt the integration time step. Since the motion of the receptors in the 2D plan is constrained by the elongated topology of dendrites (thin and long structures), receptor trajectories were decomposed along a longitudinal (the main extension of the dendrite) and a transversal direction (Figure S1A, bottom). In the custom script the x axis and y axis in (2) were the longitudinal and transversal directions, respectively. The new code estimates the diffusion coefficient (D) and the drift coefficient (μ) of the longitudinal and transversal directions from the variance (σ^2) and the mean (m) of the fitted Gaussian curves (Figure S1A) with the formulas: $\mu = m / dt$ and $D = \sigma^2 / dt$, where dt is the integration time step, m and σ can either refer to the x (longitudinal) or the y (transversal) directions. Out of 25 randomly chosen experiments, this analysis was performed those in which QD were exploring fairly linear dendrites (n = 19).

Another new custom Python program was generated to simulate inter-synaptic transitions. The Brownian motion was simulated according to the formula (2), with a dt = 50 ms (same as the experimental sampling interval) and no significant differences were

observed adopting a smaller time step (e.g., dt = 1 ms). The fastest first time passage (f-FTP) was defined as the mean value of the first percentile of the cumulative distribution (Figure S1C). The chance time was defined as the available time before the end of the recording, for the receptor to reach the target synapse after leaving a first synapse.

In order to relate the experimental first time passages to the theoretical predictions, we simulated a 1D longitudinal diffusion process (5000 simulations) with the same diffusion coefficient, chance time and inter-synaptic distance of the experiment. The intercept of the experimental first time passage on the cumulative first time passage curve of the simulated events (Figure S1D) was then reported for each experiment (Figure S1E).

Calcium imaging and pharmacology

The cell-impermeant form of Rhod-2 (50 μM) (Thermo Fisher) was added to the intracellular recording solution and allowed 15 min after reaching the whole cell configuration to diffuse into the neuron. Neurons were illuminated with 556/20nm light provided a LED source (SpectraX Lumencor, NW, USA). Rhod-2 fluorescence signal was observed with a 593/40 nm emission filter (Semrock, Italy) controlled by filter wheels mounted onto an inverted microscope (Eclipse Ti, Nikon, Japan) equipped with a 60x oil-1.4 numerical aperture (NA) immersion objective. Images were acquired every 50 ms. Changes in intracellular Ca^{2+} were elicited with brief (100 ms) or prolonged (60 s) illuminations with UV light (380nm) aimed at activating LiGluK2 or LiGluK2(Q621R), or with 300 ms depolarizations to 0 mV as indicated. Rhod-2 fluorescence over time was quantified with Metamorph software (ver. 7.8, Molecular Devices, USA) as changes in fluorescence intensity with respect to baseline ($\Delta F/F_0$). Calcium signals were corrected by photobleaching subtraction. In order to prevent the activation of VGCCs, ω-conotoxin MVIIC (2 μM, from Tocris, Italy) and nifedipine (10 μm, from Sigma, Italy) were added to the extracellular recording solution to block P/Q-, N- and L-type VGCCs, respectively. The efficacy of VGCC blockade was monitored by Ca^{2+} imaging after 5 and 8 min with respect to control values (before the application of the drugs). As reported in Figure S2E, 8 min were sufficient to achieve a complete block of P/Q-, N- and L-type VGCCs.

Immunocytochemistry

Since synaptic receptors contain different α and β subunits and obligatorily require the γ2 subunit, we immunolabelled the γ2 subunit to comprehensively target the heterogeneous populations of synaptic receptors. This approach was used to compare the expression of native and HA-GABAARs. Neurons were live labeled for 10 min at room temperature with anti-γ2 (Alomone, Israel) in the recording solution (see below) supplemented with BSA (1%) and sucrose (250mM) to prevent receptor endocytosis. After fixing with 4% paraformaldehyde (PFA) for 10 min, and blocking with BSA (1%, 10 min), neurons were incubated with fluorescence-conjugated anti rabbit secondary antibody for 45 min at room temperature. Next, neurons were permeabilized (0.2% Triton X-100 for 10 min) and sequentially incubated with the anti-vGAT antibody and fluorescence-conjugated anti mouse secondary antibody. Control experiments without the primary antibody were performed to test fluorescence signal arising from nonspecific binding of the secondary antibody. Coverslips, mounted in DAKO fluorescent mounting medium, were observed using an inverted microscope (Eclipse Ti, Nikon, Japan) equipped with a 60X oil, 1.4 NA immersion objective and a back-illuminated EMCCD camera Photometric Quantem 512S (pixel size, 160 nm). Samples were illuminated by exploiting a diode-based illumination device (Lumencor, SpectraX Light Engine, Optoprim, Italy). Images were acquired with Metamorph software (ver. 7.8, Molecular Devices, USA). The total GABAA receptor average fluorescence intensity of surface GABAA receptors in a given neuron was defined as the integrated fluorescence intensity detected in the neuron divided by the neuron pixel area and therefore expressed as au/pixel. Surface GABAARs clusters were defined as synaptic when they exhibited a juxtaposed vGAT puncta within a 2-pixel enlargement. Synaptic cluster density represents the number of synaptic clusters normalized over the neuron area, hence expressed as $\mu m^{\times 2}$. The analysis was blindly performed.

Electrophysiology and GABA uncaging

Uncaging inhibitory postsynaptic currents (uIPSCs) were recorded in the whole-cell configuration of the patch-clamp technique. External recording solution contained (in mM): 145 NaCl, 2 KCl, 2 $CaCl_2$, 2 $MgCl_2$, 10 glucose and 10 HEPES, pH 7.4. Patch pipettes, pulled from borosilicate glass capillaries (Hilgenberg, Malsfeld, Germany), had a 4-5 MΩ resistance when filled with intracellular recording solution containing (in mM): 125 KCl, 10 KGluconate, 1 EGTA, 10 HEPES and 4 MgATP, 5 sucrose (300 mOsm and pH 7.2 with KOH). Currents were recorded using Clampex 10.2 software (Molecular Devices, Sunnyvale, CA). Uncaging experiments were performed by exploiting DPNI-GABA (Tocris Bioscience), a nitroindoline-based caged compound showing high stability and uncaging efficiency, while minimizing the pharmacological block of GABAA receptors prior photolysis (Trigo et al., 2009). DPNI-GABA (1 mM) was dissolved in the extracellular solution and locally perfused through a patch pipette (2-4 μm tip diameter) by means of a pressure-based application system (10-20 psi) (Picospritzer, Parker, USA) and placed at 10 and 20 μm (x- and z axis, respectively) from the region of interest. A 378 nm diode laser (Cube 378, 16 mW, Coherent Italia, Italy) was directly coupled to the microscope objective (Olympus UPlanSApo 100X oil-1.40 NA). In order to obtain the smallest laser spot size on the sample we backfilled the objective by using a beam expander placed in the optical pathway between the laser source and the objective. The measured point spread function (PSF) of the 378 nm illumination had lateral dimension of 487 ± 55 nm (FWHM, n = 6). The laser beam was steered in the field of view by means of a galvanometric mirrors-based pointing system allowing the illumination of specific regions of interest tailored around GABAergic synapses (UGA32, Rapp OptoElectronics, Hamburg, Germany). Synchronization of optical stimulations and electrophysiological recordings was controlled with the UGA32 software interfaced with the Clampex 10.2 software (Molecular Devices, Sunnyvale, CA, USA). Currents were elicited by 500 μs light pulses at power intensity of 80-100 μW at the exit of

the objective. The laser power and the pulse duration were adjusted to minimize photo-damage and to match the IPSCs kinetics. The precision of our uncaging system was estimated by the reduction of the uIPSCs amplitude following progressive displacement of the laser spot from the center of a GABAAR synaptic cluster (FMHM = 2.34 ± 0.24 μm, n = 2, Figure S4A). Currents were obtained by the average of at least 10 traces for each condition. The stability of the patch was checked by repetitively monitoring the input and series resistance during the experiments. Cells exhibiting 10%–15% changes were excluded from the analysis. In our electrophysiology recordings, the noise was $\sigma\sim$1.5 pA. Currents were sampled at 20 kHz and digitally filtered at 3 kHz using the 700B Axopatch amplifier (Molecular Devices). Blind analysis of uncaging currents was performed with Clampfit 10.0 software (Molecular Devices, Sunnyvale, CA, USA). The analysis was blindly performed.

Cross-link (X-link) protocol

The X-link protocol restricts protein diffusion through their interaction with a primary antibody and subsequent incubation with an appropriate secondary antibody. The X-linking of HA-tagged recombinant proteins (GABAAR HA-α1 subunit and HA-NLG3) or endogenous GABAA receptors was achieved by first incubating hippocampal neurons for 10 min with an excess the anti HA primary antibody or anti GABAA receptor γ2 subunit (Alomone, Israel) and subsequently with an appropriate specie-specific secondary antibody for 10 min (Gerrow and Triller, 2014; Heine et al., 2008). After washing, neurons were used to study either GABAAR mobility or GABA uncaging synaptic currents (uIPSCs). The X-link of the γ2 subunit allowed to comprehensively target the heterogeneous populations of synaptic receptors.

Light-Activated glutamate receptors (LiGluK2)

Light-Gated Glutamate receptors (LiGluK2) have been developed by the Isacoff Lab (Volgraf et al., 2006) and consist of an engineered kainate receptor able to bind the photoswitchable tethered ligand (PTLs) maleimide-azobenzene-glutamate (MAG). In particular, the ligand glutamate is linked to azobenzene that can be reversibly photoisomerized between a *trans* and *cis* configurations in response to illumination with light at different wavelengths (380 nm and > 460 nm, respectively) (Volgraf et al., 2006). Azobenzene, in turn, is anchored to a mutated cysteine introduced into the ligand-binding domain (LBD) of GluK2 receptor through the cysteine-reactive group maleimide. Photoswitching is operated by the reversible binding of the glutamate moiety of MAG, which is presented to the ligand-binding site in the *cis* configuration and withdrawn in *trans*. The MAG molecule was kindly provided by Dr D. Trauner (The Ludwig Maximilians University of Munich) and Pau Gorostiza (Institute of Bioengineering of Catalonia, Barcelona). After dilution in DMSO, MAG was diluted in the extracellular solution to 10-50 μM (from a 10mM stock solution) and illuminated with 380 nm light to promote its accumulation of the *cis*-form, thus favoring the binding between the GluK2 glutamate binding site and the engineered cysteine in the ligand-binding domain (LBD). Hippocampal neurons were then incubated with MAG (in *cis* configuration) at 37°C for 30 min, washed, and used for recordings.

QUANTIFICATION AND STATISTICAL ANALYSIS

For each experiment quantifications and statistical details (statistical significance and test used) can be always found in the figure legends and in some instances in the main text. Unless otherwise stated, normally distributed data are presented as mean ± SEM (standard error of the mean), whereas non-normally distributed data are given as medians ± IQR (inter quartile range). For SPT experiments, n indicates the number of receptor trajectories, followed by the number of neurons observed. The number of independent neuronal cultures analyzed is also specified in each figure legend. Statistical significance was tested using Prism 5.0 Software (Graph Pad, USA). Normally distributed datasets were compared using the unpaired or paired two-tailed Student's t test (as indicated), whereas non-Gaussian datasets were tested by two-tailed unpaired non-parametric Mann-Whitney U-test or the paired Wilcoxon test. The MSD versus time curves were compared at steady state with the Student's t test. Cumulative distributions were compared with the Kolmogorov-Smirvov test using the KyPlot 5.0 software. Indications of significance corresponding to p values < 0.05 (*), $p < 0.01$ (**), $p < 0.001$ (***) and non-significant (ns) are reported in the figures and in the text.

DATA AND SOFTWARE AVAILABILITY

The custom Python scripts for quantifying diffusion coefficients based on the Gaussian fit and for simulating inter-synaptic transitions will be provided upon request to the Lead Contact.

Neuron, Volume *95*

Supplemental Information

Inter-Synaptic Lateral Diffusion of GABAA Receptors Shapes Inhibitory Synaptic Currents

Emanuela de Luca, Tiziana Ravasenga, Enrica Maria Petrini, Alice Polenghi, Thierry Nieus, Stefania Guazzi, and Andrea Barberis

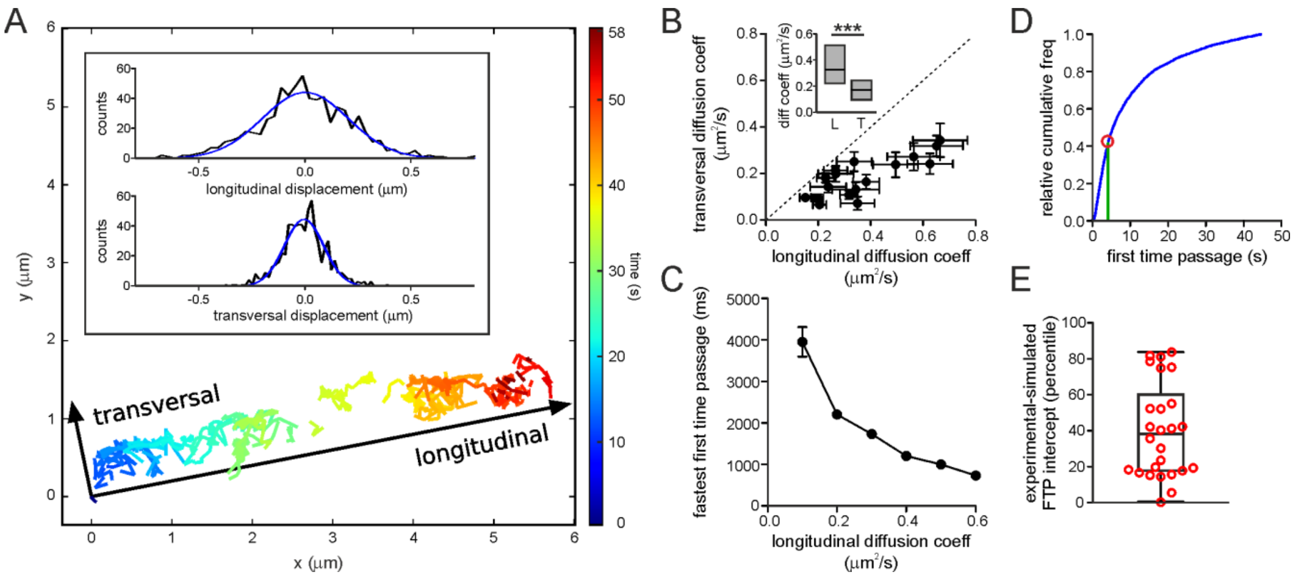

Figure S1. (Related to Figure 1) Analysis and model simulations of GABAA receptor diffusion in bounded conditions

(A) Representative trajectory of a GABAA receptor diffusing along a linear dendrite (time is represented in pseudocolors). The experimentally-recorded motion is decomposed along the longitudinal and transversal directions. Please note that blinkings are not represented, resulting in interrupted trajectory. (inset) The longitudinal and transversal displacements (black) are well fitted by a Gaussian curve (blue). Please note the much shorter displacement along the transversal direction.

(B) Longitudinal and transversal diffusion coefficients of the GABAA receptor trajectories (n=18 displacements along “mainly linear” portions of dendrites out of 25). Error bars correspond to confidence intervals. The dashed line shows identical diffusion coefficients in both the longitudinal and transversal components. Inset: Median diffusion coefficient of longitudinal (“L”) and transversal (“T”) displacements. “L”: median = 0.33 $\mu m^2 s^{-1}$, IQR=0.22÷0.67 $\mu m^2 s^{-1}$, $n_{simulated\ trajectories}$=18. “T”: median = 0.17 $\mu m^2 s^{-1}$, IQR=0.07÷0.24 $\mu m^2 s^{-1}$, $n_{simulated\ trajectories}$=18, p<0.001, Wilcoxon matched paired test U-test.

(C) Relationship between the fastest first time passage (f-FTP) and the longitudinal diffusion coefficient. The f-FTP values were obtained from the first percentile of each cumulative distribution (parameters dSYN=2 µm, TMAX=60 s). Data are presented as mean ± SEM.

(D) Representative example of FTP cumulative distribution of simulated inter-synaptic events identified in 5000 repetitions run to reproduce a single experiment (blue). In order to mimic experimental conditions, the longitudinal diffusion coefficient, the chance time and the inter-synaptic distance imposed in the simulations (see Methods) were those identified experimentally. The green line shows the inter-synaptic time quantified experimentally. The red circle highlights the intercept of the two lines, and represents the value of the percentile corresponding to the experimentally calculated inter-synaptic time. Please note that this plot refers to a single experiment.

(E) Raw data and superimposed boxplot (median and IQR range) of the “intercept values” between the inter-synaptic time and the FTP curves as described in D for all the experiments analysed (n=26). Please note that the values are nearly distributed around the 50th percentile.

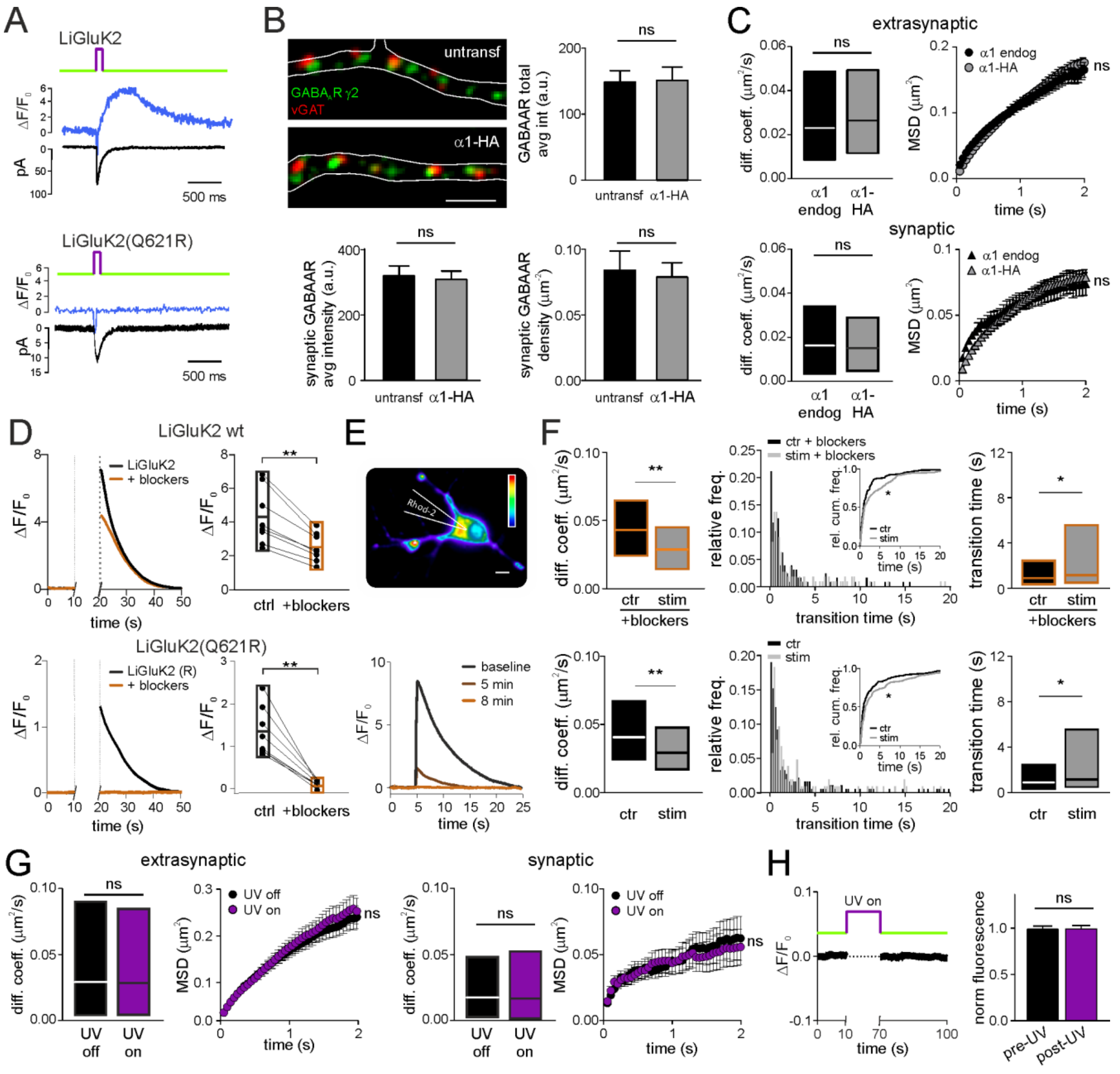

Figure S2. (Related to Figure 2) Regulation of intracellular calcium by light-gated glutamate receptor (LiGluK2) and VGCCs

(A) Representative relative changes ($\Delta F/F_0$) of Rhod-2 fluorescence (blue traces) and light-evoked currents (black traces) elicited by LiGluK2 (top) and LiGluK2(Q621R) (bottom) openings upon illumination with 380nm light. The above lines show the illumination protocol: green = 556nm and violet = 380nm. Please note that the illumination with 380 nm light (violet bars) causes an increase in intracellular calcium and consequently increases the Rhod-2 fluorescence intensity (blue traces) in LiGluK2- and not in LiGluK2(Q621R)-expressing neurons. Currents were obtained from the average of at least 10 traces.

(B) GABAAR incorporating the HA-α1 subunit show properties comparable to endogenous receptors. (top, left) Representative fluorescence images of GABAAR γ2 subunit immunoreactivity (green) along with the presynaptic marker vGAT (red) in untransfected and HA-α1–expressing neuron as indicated. Scalebar 5 μm.

(top right and bottom) Quantification of GABAARs total surface immunoreactivity and GABAARs synaptic cluster intensity and density in untransfected and HA-α1–expressing neurons (n=15 and 14, respectively, from 2 independent cultures, p>0.05, unpaired t-test).

(C) Comparable lateral mobility of endogenous and HA-tagged α1 subunits probed by SPT. (top, left) GABAAR diffusion coefficient at extrasynaptic compartments ($n_{trajectories}$: endogenous=320 in 32 neurons from 7 independent cultures; HA-α1= 609, in 54 neurons from 8 independent cultures; p>0.05, Mann–Whitney U-test). (top, right) Mean Square Displacement (MSD) vs time plots of endogenous ($n_{trajectories}$=325, in 32 neurons from 7 independent cultures) and HA-α1 tagged GABAARs ($n_{trajectories}$=609, in 54 neurons from 8 independent cultures, p>0.05, Student's t-test) at extrasynaptic compartments. (bottom, left) Diffusion coefficient of synaptic GABAARs ($n_{trajectories}$: endogenous=130, in 32 neurons from 7 independent cultures; HA-α1= 192, in 54 neurons from 8 independent cultures, p>0.05, Mann–Whitney U-test). (bottom, right) MSD vs time plots of endogenous ($n_{trajectories}$=158, in 32 neurons from 7 independent cultures) and HA-α1 GABAARs at synapses ($n_{trajectories}$=189, in 54 neurons from 8 independent cultures, p>0.05, Student's t-test).

(D) Contribution of VGCCs in the modulation of inter-synaptic displacements by LiGluK2 activation. Top: P/Q-, N- and L-type VGCCs account for ~ 40% of the calcium increase induced by LiGluK2 activation. (left) Representative examples of calcium transients following the opening of the LiGluK2 wt in control conditions (black) and after the application of P/Q-, N- and L-type VGCC blockers (ω-conotoxin MVIIC, 2μM and nifedipine, 10μM, respectively, hereafter indicated as "VGCCs blockers") (orange). (right) Matched peak calcium transients induced by LiGluK2 wt openings before (black) and after (orange) the application of VGCC blockers (n=8 neurons, p<0.01, paired Wilcoxon test). Bottom: Upon the activation of LiGluK2(Q621R), the calcium increase is ~ 70% less than that mediated by LiGluK2 wt, and is mainly accounted for by P/Q-, N- and L-type voltage-gated calcium channels. (left) Representative traces of calcium transients following the opening of the LiGluK2(Q621R) in control conditions (black) and after the application of the VGCC blockers (orange). (right) Matched peak calcium transients induced by LiGluK2(Q621R) opening before (black) and after (orange) the VGCCs block (n=8 neurons, P<0.01, paired Wilcoxon test).

(E) Pharmacological block of VGCCs. (top) Representative cultured hippocampal neuron expressing LiGluK2 loaded with Rhod-2 (shown in pseudocolors). Scale bar 5 μm. (bottom) Representative calcium transients evoked by cell depolarization (300ms, 0mV) in control condition (black), 5 minutes (brown) and 8 minutes (orange) after the application VGCC blockers. Note that at 5 minutes the calcium increase is only partially blocked, whereas it is completely abolished after 8 minutes.

(F) During LiGluK2 activation, VGCCs do not play a major role in the modulation of GABAA receptor inter-synaptic mobility. Top: Modulation of HA-GABAAR inter-synaptic transitions by LiGluK2 activation in the presence of VGCC blockers. (left) Inter-synaptic HA-GABAAR diffusion coefficient in the presence of VGCC blockers before (ctr, $n_{trajectories}$=72) and upon LiGluK2 activation ($n_{trajectories}$=63; in 17 neurons from 5 independent cultures, p<0.01, Mann–Whitney U-test). (middle) Histogram and cumulative distribution (inset) of inter-synaptic displacement time in the control (black) and during LiGluK2 activation (gray) upon VGCCs blockade. (n=129 and 110, respectively, in 17 neurons from 5 independent cultures, p<0.05, Kolmogorov Smirnov test). (right) HA-GABAAR median and IQR of inter-synaptic transition time in control (n=129) and upon stimulation with LiGluK2 in the presence of VGCC blockers (n= 110; p<0.05, Mann–Whitney U-test). Bottom: Same as above, without VGCC blockers. (left) Diffusion coefficient of inter-synaptic HA-GABAARs before (ctr, $n_{trajectories}$=97) and during LiGluK2 activation (stim, $n_{trajectories}$=66; p<0.01, Mann–Whitney U-test; 18

neurons from 5 independent cultures). (middle) Histogram and cumulative distribution (inset) of inter-synaptic displacement time in the control (black) and during LiGluK2 activation (gray). (n=185 and 104, respectively, in 18 neurons from 5 independent cultures, $p<0.05$, Kolmogorov Smirnov test). (right) HA-GABAAR median and IQR of inter-synaptic transition time in control (n=185) and upon stimulation with LiGluK2 (n= 104; $p<0.05$, Mann–Whitney U-test; 18 neurons from 5 independent cultures).

(G) UV illumination does not alter GABAAR lateral mobility. Diffusion coefficient (left) and MSD vs time plots (right) of HA-GABAAR illuminated with 490nm (UV off, black) or with 380nm (UV on, violet) light at extrasynaptic and synaptic compartments as indicated. Extrasynaptic diffusion coefficient, $n_{trajectories}$: UV off=263; UV on= 232, in 12 neurons from 2 independent cultures; $p>0.05$, Mann–Whitney U-test. MSD of extrasynaptic HA-GABAAR, $n_{trajectories}$: UV off=282, UV on=258, in 12 neurons from 2 independent cultures; $p>0.05$, Student's t-test. Diffusion coefficient of synaptic HA-GABAAR, $n_{trajectories}$: UV off=61; UV on = 61, in 12 neurons from 2 independent cultures, $p>0.05$, Mann–Whitney U-test. MSD of synaptic HA-GABAARs, $n_{trajectories}$: UV off=66; UV on=66, in 12 neurons from 2 independent cultures, $p>0.05$, Student's t-test.

(H) UV illumination does not alter intracellular calcium. (left) Representative relative change in Rhod-2 fluorescence ($\Delta F/F_0$) elicited by 60 s UV illumination of an untransfected neuron. The green and violet lines above show the illumination pattern as in panel A. (right) Normalized Rhod-2 fluorescence quantified before and after 60 s UV illumination (n=12, $p>0.05$, paired t test).

Unless otherwise stated, data are presented as mean values ± SEM. The boxplots indicate the median and interquartile range (IQR). Relative changes in Rhod-2 fluorescence are expressed as percentage units. ns, non significant; *, $p<0.05$; **, $p<0.01$.

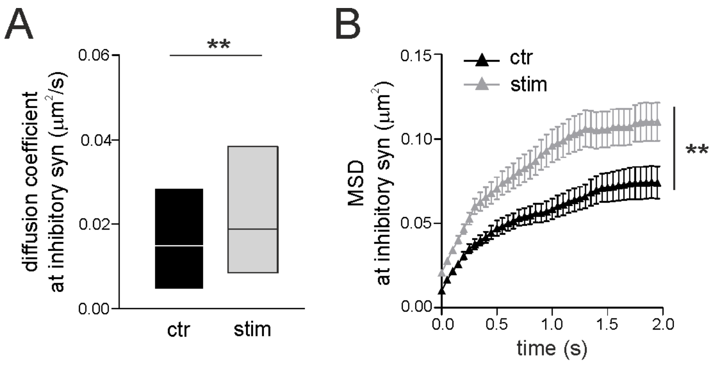

Figure S3. (Related to Figure 3) LiGluK2 activation modulates GABAAR diffusion at inhibitory synapses

(A) Diffusion coefficient of synaptic GABAAR for control (ctr) and 1 min LiGluK2 activation (stim) (median=0.014 $\mu m^2 s^{-1}$, IQR=0.006-0.033 $\mu m^2 s^{-1}$, $n_{trajectories}$=107; and median=0.021 $\mu m^2 s^{-1}$ IQR=0.010-0.046 $\mu m^2 s^{-1}$, $n_{trajectories}$=128, respectively, $p<0.01$; Mann–Whitney U-test; 28 neurons from 7 independent cultures).

(B) MSD versus time plot of GABAARs diffusing at inhibitory synapses (steady state: ctrl=0.075±0.009 μm^2, $n_{trajectories}$ 107 and stim=0.110±0.011 μm^2, $n_{trajectories}$ 128; 28 neurons from 7 independent hippocampal cultures, $p<0.01$, Student's t-test at steady state).

Data are presented as mean values ± SEM. The boxplots indicate the median and IQRs. **, $p<0.01$.

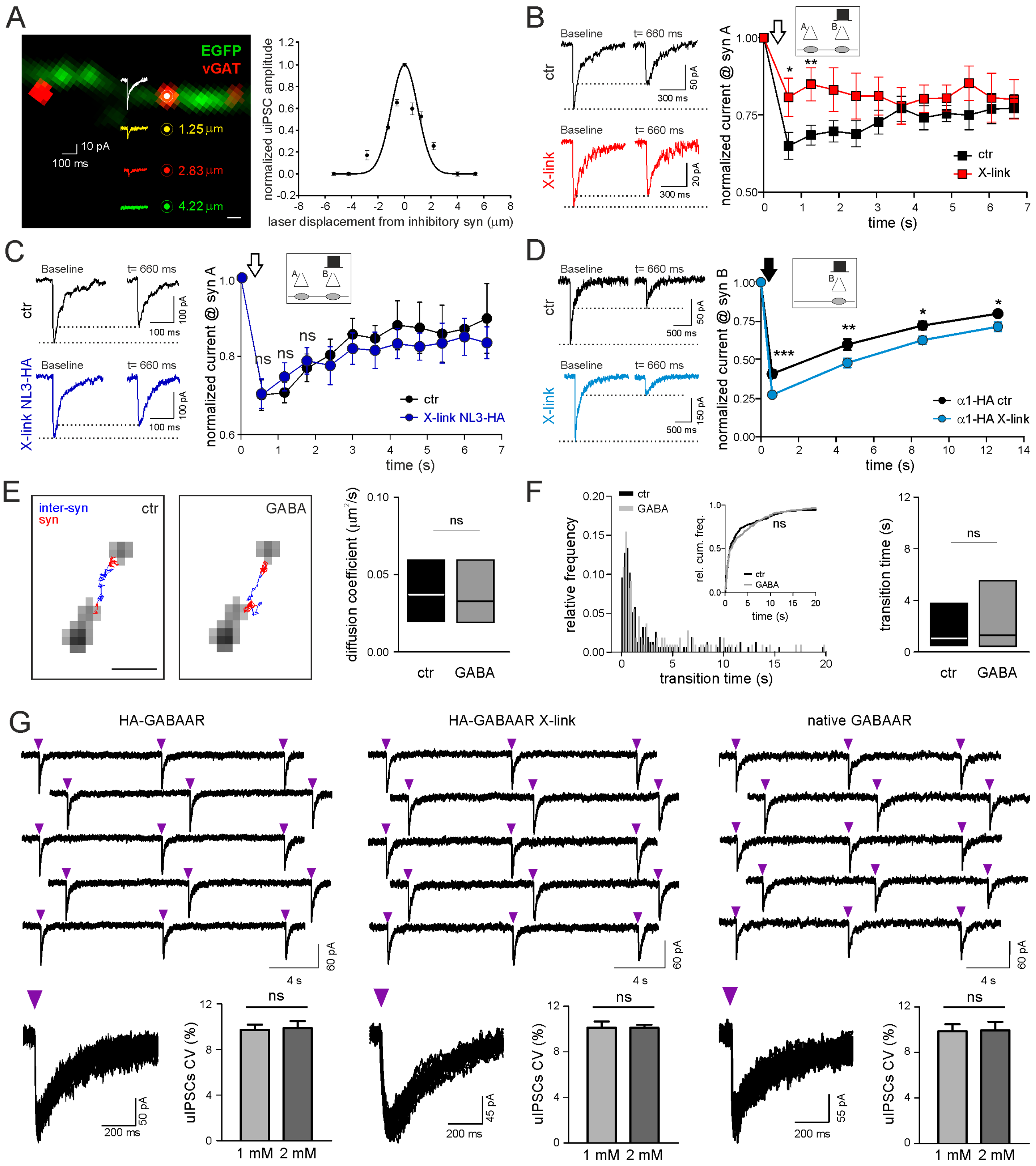

Figure S4. (Related to Figure 4) GABAA receptor desensitization by GABA uncaging and functional implications

(A) (left) Representative image of a EGFP-transfected neuron (green) with GABAergic synapses identified by live labelling with anti-vGAT-Oyster550 antibody (red). Scale bar: 500 nm. uIPSCs were elicited by laser pulses delivered in the synapse center (white) and progressively displaced away from the synapse (yellow: 1.25 µm; red: 2.83 µm; green: 4.22 µm). (right) Summary of the relationship between uIPSCs amplitude and the laser spot position with respect to the synapse center. The measured full width at half maximum (FWHM) was 2.34±0.24 µm (n=2).

(B) The inter-synaptic diffusion of endogenous GABAA receptors modulates inhibitory synaptic responses. (left) Representative traces of uIPSCs mediated by endogenous GABAA receptors in control (ctr, black) and after the X-link protocol (X-link, red), before and 660 ms after the delivery of a UV laser train pulse at an adjacent inhibitory synapse, according to the protocol shown in Figure 4A and schematized in the inset on the right. Dashed lines show the reduction in uIPSCs current amplitude before and after the train (i.e., desensitization). (right). Normalized recovery over time of uIPSCs recorded at synapse "A" after delivery of the UV laser train pulse at synapse "B" (arrow) in control (ctr, black, n= 18, from 2 independent neuronal cultures) and after the X-link protocol in untransfected neurons (X-link, red, n= 16, from 2 independent neuronal cultures). At 660 ms: ctr=0.65±0.04, n=18; X-link=0.81±0.06, n=16; p<0.05. unpaired t test.

(C) The X-link protocol does not affect the recovery of uncaging currents. (Left) Representative traces of uIPSCs recorded in control (ctr) and in Neuroligin3 X-link protocol (NL3) before (baseline) and 660 ms after the UV laser train of stimuli at an adjacent synapse. Dashed lines show the reduction in uIPSCs current amplitude before and after the train (i.e., desensitization). (right) Normalized uncaging current amplitude recovery of synapse B (see protocol schematization in the inset) over time after the application of the UV laser train at synapse A (arrow) in control (ctr-black, n=12) and upon Neuroligin3 X-link (NL3 blue n=14). Please note that the X-link protocol directed to the synaptic protein NL3 does not affect the time course of the uncaging current amplitude recovery (time point 660 ms: ctr=0.71±0.03, n_{values}=12 from 11 neurons and 5 independent hippocampal cultures; NL3=0.75±0.04, n_{values}=14 from 7 neurons and 3 independent hippocampal cultures; p>0.05, Mann–Whitney U-test).

(D) Repetitive GABA uncaging at inhibitory synapses renders GABAAR into the desensitized state. (left) Representative uncaging currents elicited before (left) and 660 ms after (right) the delivery of the UV laser train pulse in control conditions (ctr, black) and upon X-link (light blue). Please note that in these experiments the train is delivered at the same synapse tested (see protocol schematization in the inset). Dashed lines show the reduction in uIPSCs current amplitude before and after the train (i.e., desensitization). (right) Normalized uncaging current amplitude over time after delivery of the UV laser train pulse (plain arrow) in control (ctr, black, n= 8 from 3 independent neuronal cultures) and upon X-link (X-link, light blue, n= 14 from 4 independent neuronal cultures). At 660 ms: ctr=0.41±0.03, n=8; X-link=0.27±0.02, n=14; p< 0.001, Mann–Whitney U-test.

(E) Inter-synaptic displacements of desensitized GABAAR. (left) Reconstructed synaptic (red) and inter-synaptic (blue) trajectories of the same HA-GABAA receptor in control conditions and upon replacement of the recording solution with GABA 100 μM, a concentration that, being more than 5 times the GABA EC50, is expected to promote massive GABAA receptor desensitization. Live-immunolabelling of vGAT at inhibitory synapses is shown in gray. Scalebar 1 μm. (right) Diffusion coefficient of inter-synaptic HA-GABAAR in control (median=0.037 $\mu m^2 s^{-1}$, IQR=0.020-0.059, $n_{trajectories}$=95; GABA 100 μM (GABA), median=0.033 $\mu m^2 s^{-1}$, IQR=0.019-0.060, $n_{trajectories}$=65; 19 neurons from 5 independent cultures; p>0.05, Mann–Whitney U-test).

(F) (left) Histogram and cumulative distribution (inset) of HA-GABAAR inter-synaptic displacement time in the control (black, n=153) and upon solution exchange with GABA 100 μM (gray, n=109), p>0.05, Kolmogorov Smirnov test. (right) Inter-synaptic transition time of desensitized HA-GABAAR. Control: Median=1.05 s; IQR=0.50-3.78 s, n=153; GABA: Median=1.30 s; IQR=0.45-5.52 s, n=109; 19 neurons from 5 independent cultures; p>0.05, Mann–Whitney U-test. (G) Saturation of inhibitory synapses by GABA uncaging pulses. Top. Representative uncaging experiments aimed at probing synaptic saturation by quantification of synaptic

uIPSCs variability in neurons expressing HA-GABAAR (left), HA-GABAAR upon X-link (middle) and native GABAA receptors (right). Laser pulses were delivered every 10 s to uncage GABA 1 mM (violet arrowheads) at inhibitory synapses. (Bottom) Superimposed uIPSCs recorded in the experiment shown above and mean coefficient of variation (CV) of uIPSCs elicited by the laser photolysis of caged GABA 1 mM (9.73±0.47 %, n= 5) and 2 mM (9.88±0.62 %, n= 4, p>0.05, unpaired t test) in the indicated conditions, i.e. HA-GABAAR (left), HA-GABAAR upon X-link (middle) and native GABAA receptors (right). The unaffected CV values with a double concentration of caged GABA (2 mM) indicate the saturation of inhibitory synapses challenged with the uncaging pulses. In neuron expressing HA-GABAAR: $CV_{GABA\ 1\ mM}$ = 9.73±0.47 % (n= 5); $CV_{GABA\ 2\ mM}$ = 9.88±0.62 % (n= 4), p>0.05, unpaired t test. In neurons expressing HA-GABAAR upon receptor X-link: $CV_{GABA\ 1mM}$ = 10.13±0.52 (n=5); $CV_{GABA\ 2mM}$ = 10.11±0.27 (n=3), p>0.05, unpaired t test. In untransfected neurons expressing only native GABAARs: $CV_{GABA\ 1mM}$ = 9.87±0.62 (n=5); $CV_{GABA\ 2mM}$ = 9.95±0.75 (n=5), p>0.05, unpaired t test.

Unless otherwise stated, data are presented as mean values ± SEM. The boxplots indicate the median and IQRs. ns, non significant; *, p<0.05; **, p<0.01. ***, p<0.001.
